# Exploded view of higher order G-quadruplex structures through click-chemistry assisted single-molecule mechanical unfolding

**DOI:** 10.1093/nar/gkv1326

**Published:** 2015-11-30

**Authors:** Sangeetha Selvam, Zhongbo Yu, Hanbin Mao

**Affiliations:** Department of Chemistry and Biochemistry, Kent State University, Kent, OH 44242, USA

## Abstract

Due to the long-range nature of high-order interactions between distal components in a biomolecule, transition dynamics of tertiary structures is often too complex to profile using conventional methods. Inspired by the exploded view in mechanical drawing, here, we used laser tweezers to mechanically dissect high-order DNA structures into two constituting G-quadruplexes in the promoter of the human telomerase reverse transcriptase (hTERT) gene. Assisted with click-chemistry coupling, we sandwiched one G-quadruplex with two dsDNA handles while leaving the other unit free. Mechanical unfolding through these handles revealed transition dynamics of the targeted quadruplex in a native environment, which is named as native mechanical segmentation (NMS). Comparison between unfolding of an NMS construct and that of truncated G-quadruplex constructs revealed a quadruplex–quadruplex interaction with 2 kcal/mol stabilization energy. After mechanically targeting the two G-quadruplexes together, the same interaction was observed during the first unfolding step. The unfolding then proceeded through disrupting the weaker G-quadruplex at the 5′-end, followed by the stronger G-quadruplex at the 3′-end via various intermediates. Such a pecking order in unfolding well reflects the hierarchical nature of nucleic acid structures. With surgery-like precisions, we anticipate this NMS approach offers unprecedented perspective to decipher dynamic transitions in complex biomacromolecules.

## INTRODUCTION

Unlike proteins or RNAs, which demonstrate abundant examples of tertiary interactions, higher order DNA structures are not well known until G-quadruplexes start to attract research attention after their *in vivo* formation and biological activities have been recognized (1,2). A G-quadruplex comprises G-quartets stacking into a columnar structure through cation coordination and π–π interactions. Each G-quartet consists of four guanines interconnected to each other by Hoogsteen bonds (3).

A G-quadruplex requires at least four G-tracts to fold. Computerized search has indicated plenty of G-quadruplex hosting regions in human genome (4,5). In many regions, multiple G-tracts exist, which allows the folding of more than one G-quadruplex (6–9). This brings an opportunity for neighboring G-quadruplexes to interact. Indeed, experiments have confirmed quadruplex–quadruplex interaction (QQI) in adjacent G-quadruplexes (6,9–12). Such a discovery is of high significance to develop drugs with desired G-quadruplex specificity.

Due to the biological activities demonstrated by G-quadruplex, it becomes a viable approach to regulate these biological functions via G-quadruplex interacting ligands. It has been successfully demonstrated that ligands can differentiate between G-quadruplex structures and duplex DNA (13,14), which reduces side effects originated from non-discriminative binding of ligands to the DNA duplex. However, ligand specificity among different G-quadruplexes is so far problematic to achieve. The interaction between G-quadruplex units is expected to generate new targets through which specific ligands can be designed.

Nevertheless, it is rather challenging to identify and interrogate tertiary interactions, such as QQI, in macromolecules. First, tertiary interactions are of long-range in nature. Unlike the localized primary or secondary structures, long-range interaction involves structural components located in distance. Pinpoint these interactions require comprehensive knowledge of both local and global architectures of a macromolecule. Second, due to the very nature of tertiary interactions, macromolecules hosting these interactions are often large. Conventional structural determination methods such as X-ray and nuclear magnetic resonance have compromised capacity to probe large molecules.

Inspired by exploded drawing of components in a mechanical device, here, we developed a new approach to localize and characterize tertiary interactions in biomacromolecules by breaking down the entire structure into individual components with an exploded perspective. As a proof of concept, we demonstrated this method in a DNA sequence located upstream (–22 to –90) of the transcription start site of the human telomerase reverse transcriptase (hTERT) gene, or hTERT1–12.

**Figure 1.**
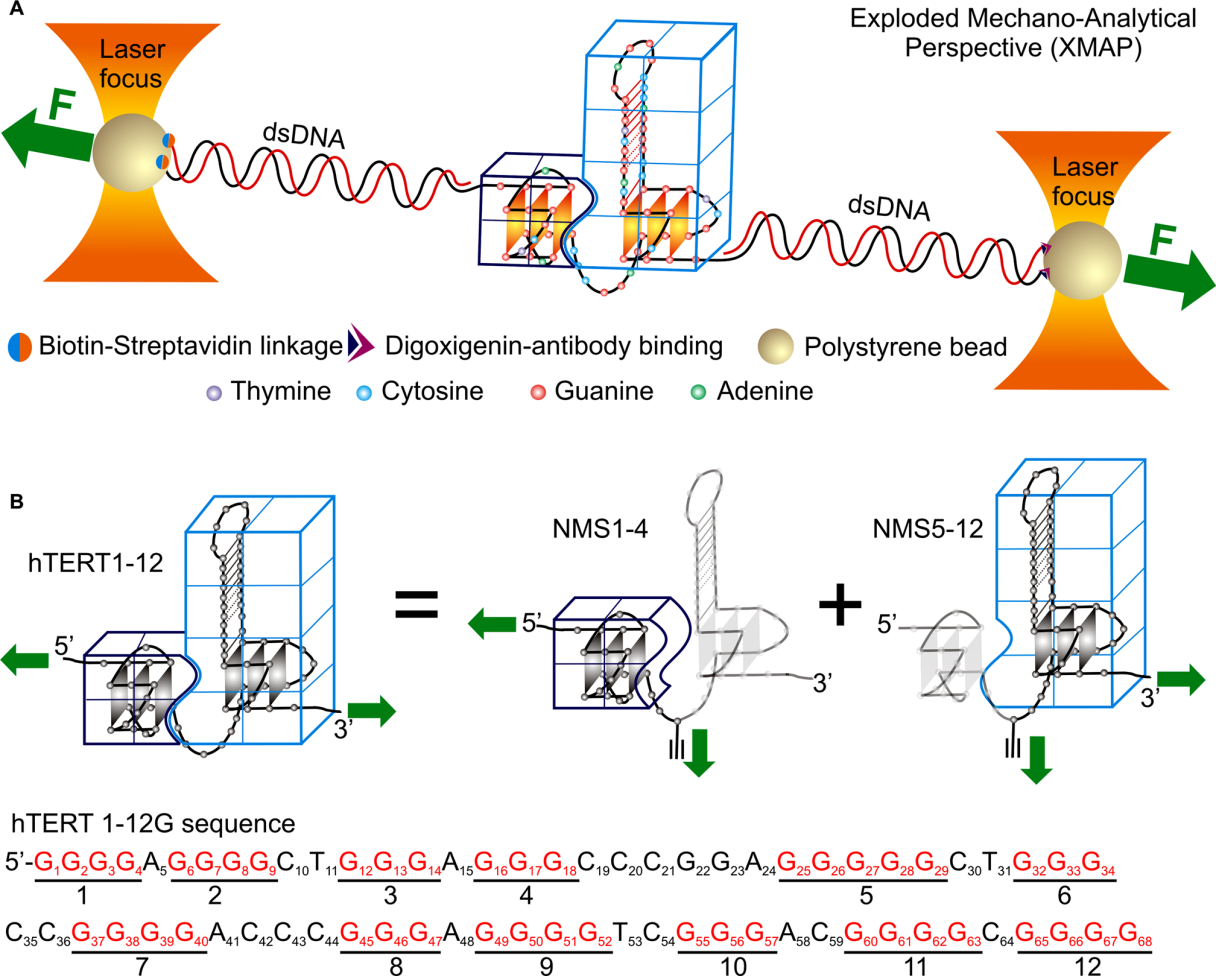
(**A**) Schematic drawing of the mechanochemical unfolding of the two adjacent hTERT G-quadruplexes using optical tweezers. (**B**) The higher order structure between the two G-quadruplexes in the hTERT1–12 construct is depicted by the two dissected G-quadruplexes (the 5′-end G-quadruplex [NMS1–4] and the 3′-end G-quadruplex [NMS5–12]) from an exploded perspective. Only the bold regions depicted in frames are targeted during mechanochemical unfolding whereas the light-colored regions do not experience forces. The green solid arrows indicate the direction at which the force is applied to the construct through the dsDNA handles. Sequence of the hTERT 1–12 G-tracts is shown at the bottom.

This region consists of 12 G-tracts capable of forming two G-quadruplex structures in the 1^st^–4^th^ and 5^th^–12^th^ G-tracts, respectively (Figure 1). G-quadruplexes in the hTERT promoter are proposed to control the expression of the telomerase (10,15), which has elevated concentration in majority of cancer cells. The potential interaction between the two G-quadruplexes may offer another level of modulation for the telomerase expression. Although such a tertiary interaction has been proposed (10), no direct evidence has been presented.

Since higher order QQI has been proposed to occur between two G-quadruplexes (6,10,11), our strategy dissects the entire DNA structure into individual G-quadruplexes followed by separate characterization of each quadruplex unit. To probe the high-order communication between quadruplexes, the dissection should avoid disrupting tertiary interactions. The truncation strategy in which a part of the structure is deleted is, therefore, not appropriate. Instead, we rely on click chemistry (16,17) to achieve this goal. By selectively attaching a terminal alkyne group to a specific residue between the two hTERT G-quadruplexes, we were able to introduce a dsDNA handle with a terminal azide group in this region through the Cu(I)-catalyzed azide-alkyne cycloaddition (CuAAC) reaction (16,17). Next, another DNA handle can be attached to either the 5′- or 3′-end of the hTERT sequence that contains the full 12 G-tracts. As a result, a desired structural segment that contains one G-quadruplex can be mechanically targeted by applying force via the two DNA handles in a laser-tweezers instrument. Since the rest of the structure does not experience any mechanical force, this strategy only investigates the desired structural segment while keeping the remainder of the structure mechanically secluded. We name this strategy as native mechanical segmentation (NMS) to reflect the surgery-like precision for the mechanical dissection of a native biomolecule.

With this strategy, we found small unfolding transitions consistent with the disruption of a higher order interaction between the two hTERT G-quadruplexes in the DNA construct in which either the 1–4 or the 5–12 G-tracts are mechanically targeted. The same interaction also existed in the construct that has all 12 G-tracts mechanically targeted. Scrutinizing on the two mechanically segmented DNA constructs suggests that the higher order interaction is likely due to the stacking between the two G-quadruplexes with a stabilizing free energy of 2.0 ± 0.4 kcal/mol. In addition, we revealed that unfolding pathways of the whole structure can be pieced together by those presented in each G-quadruplex, confirming the hierarchical organization of tertiary DNA structures. Although complex DNA structures have been investigated here through dichotomizing individual structural domains with an exploded mechano-analytical perspective (XMAP), the XMAP approach can be readily extended to interrogate other biomacromolecules such as RNAs and proteins.

## MATERIALS AND METHODS

### Materials

All DNA fragments unless specifically mentioned were purchased from Integrated DNA Technologies (Coralville, IA, USA). The DNA fragments were purified by 8% polyacrylamide gel electrophoresis. The alkyne modified DNA sequence used in the NMS1–4 and NMS5–12 constructs was purchased from Baseclick GmbH, Germany. Chemicals used in this work were purchased from VWR (West Chester, PA, USA) with purity >95% and used directly without further purification. All restriction enzymes were purchased from New England Biolabs Inc. (Ipswich, MA, USA). Streptavidin coated polystyrene particles (1.87 μm diameter) and digoxigenin-antibody coated polystyrene particles (2.1 μm diameter) were purchased from Spherotech Inc. (Lake Forest, IL, USA).

### Circular dichroism (CD) measurements

Circular dichroism spectrum was measured using Jasco-810 spectropolarimeter (JASCO Inc., Easton, MD, USA). The oligonucleotide (ODN 186, sequence see Supplementary Table S1) sample solution (5 μM) was prepared in a 10 mM Tris buffer (pH 7.4) with 100 mM KCl. The spectrum was recorded at 25°C in the wavelength range of 200–360 nm using a quartz cuvette with 1 mm path length at a scanning speed of 50 nm/min. Three measurements were recorded for the DNA sample and averaged. Prior to the CD measurement, the sample was denatured at 95°C for 10 min and cooled to room temperature in 2 h. CD spectrum of ODN 186 showed peaks at 210 nm and 265 nm, a shoulder at 290 nm and a valley at 240 nm (Supplementary Figure S1). These features were identical with those from the wild type sequence (10) (see Figure 4A in reference (10)), confirming conformations of G-quadruplexes were not altered after the alkyne modification of the C21 residue.

### Synthesis of DNA constructs for single-molecule analysis

All the DNA constructs synthesized for this project comprise of a sequence of interest sandwiched between two long dsDNA handles. One of the dsDNA handles (2028-bp in length) was modified with biotin at the 5′-end (introduced through a biotinylated polymerase chain reaction (PCR) primer using a plasmid pBR322 template (site 629^th^–2961^st^)) and the other handle was 2690-bp in length with multiple copies of digoxigenin introduced by terminal transferase at the 3′-end of the plasmid pEGFP fragment (site 620^th^–2663^rd^, purified from SacI and EagI restriction enzyme digestions) (18). These modifications allowed the attachment of the DNA construct to the two types of protein coated polystyrene particles (see Materials) through biotin-streptavidin linkage and digoxigenin-anti-digoxigenin linkage, respectively.

The hTERT1–12, truncated1–4 and truncated1–4 + 7-nt constructs were prepared following the procedure shown in Supplementary Figure S2. The sequence of interest (SOI) in each of these constructs was flanked by two 26-nt sequences (see Supplementary Table S1 for the sequence). Two different oligonucleotides, which were complementary to the flanking regions of the SOI, were annealed, followed by the ligation with the two long dsDNA handles. In the case of the NMS1–4 or the NMS5–12 construct, the end that was not labeled with biotin in the 2028-bp dsDNA handle was modified with an azide group during the PCR preparation using a 5′- azide modified primer (site 629^th^–2961^th^ of the plasmid pBR322 was used as the PCR template).

The hTERT1–12 fragment with a terminal alkyne group labeled at the residue C21 (ODN 186, see Supplementary Table S1 for the sequence: the modified cytosine is marked with an asterisk) was used in the NMS1–4 or the NMS5–12 construct. The 3′-end of the hTERT 1–12 G-tracts region was connected to a 29-nt flanking sequence. For the synthesis of the NMS5–12 construct, the oligonucleotides complementary to the flanking sequence at the 3′-end were annealed at 96°C for 10 min, followed by slow cooling to 25°C for about 5 h. During annealing, an EagI restriction site was generated at the 3′-end of the fragment, which was used to ligate with the 2690-bp dsDNA handle prepared above (similar to step B in Supplementary Figure S3 and the gel image corresponding to this step was shown in Supplementary Figure S4B). Following the DNA ligation, the azide-modified 2028-bp handle with biotin labeled at the other end was linked to the alkyne group at the C21 position of the hTERT1–12 fragment through the CuAAC reaction (similar to step C in Supplementary Figure S3; see Supplementary Figure S4D for the gel image of the reaction) (16,17). For the click chemistry coupling, the respective DNA sequences were mixed in the click reaction medium (1:2 ratio of 0.1 M CuBr / 0.1 M Tris(benzyltriazolylmethyl)amine in 3:1 DMSO/t-BuOH) and incubated overnight in absence of light. As the reaction mixture contained copper ions, 25 mM EDTA was added to remove the free ions after the click reaction. The DNA sample was purified by ethanol precipitation prior to single-molecule mechanochemical analysis.

For the synthesis of the NMS1–4 construct, the same hTERT1–12 fragment whose C21 was labeled with the terminal alkyne (ODN 186, see Supplementary Table S1) was used to attach two dsDNA handles that flanked the hTERT 1–4 G-tracts. This task required a flanking sequence on the 5′-end of the hTERT1–12 fragment. To introduce this sequence (ODN 187.1, see Supplementary Table S1 for the sequence), we developed a new synthetic strategy, KELPS (Kinetic Enrichment of Ligation via Product Selection, see main text and Supplementary Figure S3). First, a splint ligation (19) by T4 DNA ligase was carried out in which a DNA splint (SSd003.2) was hybridized to the 5′-end of the ODN 186 and 3′-end of the ODN 187.1 (step A1 in Supplementary Figure S3). To remove the DNA splint (SSd003.2) that was partially hybridized with the G-rich region in the hTERT1–12 fragment, we used another oligonucleotide (SSd003.3). This sequence was designed to have a toehold section at the 5′-end that can recognize the 5′-end flanking sequence of the hTERT1–12 while generating an EagI overhang. However, it would not interfere with the G-rich region of the hTERT1–12 fragment. To fully replace the oligonucleotide SSd003.2, 50 times excess of the SSd003.3 was used at 96°C for 10 min, followed by immediate cooling to 4°C (step A2 in Supplementary Figure S3). After replacement reaction, the above mentioned 2690-bp DNA handle was ligated to the modified hTERT1–12 fragment (step A3) through the newly generated flanking sequence at the 5′-end (step B in Supplementary Figure S3; refer Supplementary Figure S4A for gel image). Finally, the NMS1–4 construct was obtained by coupling of the azide-modified 2028-bp DNA handle to the alkyne modified hTERT-2690-bp DNA complex through the CuAAC reaction discussed above (step C in Supplementary Figure S3; gel image corresponding to this click reaction was shown in Supplementary Figure S4B). After the click reaction, the reaction mixtures were treated with ethylenediaminetetraacetic acid (EDTA) in equimolar ratio to Cu (II) ions added during the click reaction to remove the copper. Later, this solution was centrifuged at 1300 rpm for 5 min to collect the supernatant. The supernatant containing the DNA was purified by ethanol precipitation. The pellet was then directly used for the mechanical unfolding experiments after reconstitution with desired buffer. Although there were reactants in the pellet (see Supplementary Figure S4C,D), they did not affect mechanical unfolding experiments since they could not be tethered between the two optically trapped beads, which requires the labeling of both biotin or digoxigenin at the two ends of a DNA construct.

### Single-molecule force ramping experiments

The detailed description of the home-made dual-trap optical tweezers instrument for single-molecule force spectroscopy experiments has been reported elsewhere (20). Briefly, a laser beam from a single laser source (1064 nm, CW mode, 4 W, BL-106C, Spectra-physics) was split into two polarized beams using a polarized beam splitter. The foci of the two laser beams served as two optical traps. The movement of that optical trap was controlled by a steerable mirror (Nano-MTA, Mad City Labs, Madison, WI, USA) at a back focal plane of the focusing objective (Nikon CFI0 Plan-Apochromat 60x, NA 1.2, water immersion, working distance ≈320 μm). The positions of the two laser traps were detected using position-sensitive photodetectors (DL100, Pacific Silicon Sensor) and the force exerted on the traps were calibrated by thermal motion measurement. A three-channel microfluidic chamber was used for the single-molecule assays. The middle channel serving as the reaction center was connected to the top or bottom channel via a micrometer-sized glass pipette (King Precison Glass Inc., Claremont, CA, USA). The streptavidin and digoxigenin antibody coated polystyrene beads were introduced to the central channel via the pipettes from the top and bottom channels, respectively.

The synthesized DNA construct modified with either biotin or digoxigenin on each end was incubated with digoxigenin-antibody coated polystyrene particles (2.1 μm, Spherotech Inc.) for 1 h. During the incubation, the linkage between the digoxigenin in the DNA construct and the digoxigenin-antibody on the bead surface was established. A DNA tethered bead and a streptavidin coated polystyrene particle were trapped in the two laser foci, respectively. Upon moving one of the laser foci toward the other, the biotin-modified end of the DNA was linked to the streptavidin coated bead, accomplishing the tether of the DNA to the two trapped polystyrene beads. The force-ramping experiments were then carried out by moving the two traps toward, or away from, each other. The loading rate of the stretching and relaxing a DNA tether was kept at 5.5 pN/s. The single-molecule nature was determined by a characteristic saw-tooth plateau observed at 65 pN on stretching the DNA tethered between the two beads. All single-molecule analysis reported was performed in 10 mM Tris buffers (pH 7.4) supplemented with 100 mM K^+^ at room temperature (23°C).

### Determination of unfolding pathways

Stretching of the hTERT DNA constructs generated more than one feature in most of the *F-X* traces. The folded structure corresponding to each unfolding event was assigned based on the change-in-contour-length (Δ*L*, see below and Supporting Information for calculation) and the order of occurrence during stretching. For accurate assignment of different species, only the molecules showing the Δ*L* for the fully folded structure were selected for analyses (68 nts or 25 nm for the hTERT1–12 construct and 48 nts or 18 nm for the NMS5–12 construct). About 11% and 31% of the total traces showed unfolding of the fully folded structure to fully unfolded sequence in one step for the hTERT1–12 and NMS5–12 constructs respectively. To assign the unfolding intermediates, first, each feature was categorized into different Δ*L* bins with a bin width of 4 nm. This value was determined based on 3 standard deviations of the Δ*L* measurements. The categorization was followed by ranking each feature in a trace according to the order of occurrence. Many traces contained features with almost identical Δ*L* between two transitions. By this ranking method, respective folded structures and their orders of occurrence can be deduced after converting the Δ*L* of a particular transition to the number of nucleotides using Equation S1 shown in Supporting Information. The population of each folded state was determined by the total number of transitions determined for that state (see Supplementary Table S2 for detailed statistics). The percentage of each transition in an unfolding pathway was determined by the total number of transitions originated from that particular state.

## RESULTS AND DISCUSSION

### The exploded mechano-analytical perspective (XMAP) for DNA tertiary structures

It has been proposed that the two G-quadruplexes formed in the hTERT 1–12 sequence may interact through the stacking of terminal G-quartets from individual G-quadruplex units (10). Such stacking brings two G-quadruplexes closer, it is therefore anticipated that the oligonucleotides in the inter-quadruplex region should remain in a compact form. Removal of the tertiary interaction between the two G-quadruplexes would stretch out this region, generating an observable transition in force-extension (*F-X*) curves. Our exploded mechano-analytical perspective (XMAP) on the tertiary interaction is designed to focus on this region (Figure 1). First, we plan to dissect this region into two segments by modifying an inter-quadruplex cytosine (position C21, see Table 1) with a terminal alkyne (17). An azide-labeled dsDNA handle can then be attached to the cytosine residue through Cu(I)-catalyzed click chemistry reaction (16). Depending on the specific G-quadruplex (Figure 1B) for mechanical targeting, we can attach another dsDNA handle either to the 5′- or 3′-end of the hTERT 1–12 template. A total of three hTERT DNA constructs will be generated (Figure 1). Sequence hTERT1–12 has the two dsDNA handles flanking the entire 1^st^–12^th^ hTERT G-tracts; sequence NMS1–4 has the two handles flanking the 1^st^–4^th^ G-tracts; while the NMS5–12 construct has the dsDNA handles sandwiching the 5^th^–12^th^ G-tracts. Mechanical unfolding on each of the three sequences can then be carried out and discussed in the following sections.

**Table 1.** Sequence of interest from hTERT 1–12 region studied under the various constructs used in this study

DNA construct	Sequences of interest from the hTERT 1–12 region
hTERT 1–12	5′-**G_1_G_2_G_3_G_4_A_5_G_6_G_7_G_8_G_9_C_10_T_11_G_12_G_13_G_14_A_15_G_16_G_17_G_18_C_19_C_20_C_21_G_22_G_23_A_24_G_25_G_26_G_27_G_28_G_29_ C_30_T_31_G_32_G_33_G_34_C_35_C_36_G_37_G_38_G_39_G_40_A_41_C_42_C_43_C_44_G_45_G_46_G_47_A_48_G_49_G_50_G_51_G_52_T_53_C_54_G_55_G_56_G_57_ A_58_C_59_G_60_G_61_G_62_G_63_C_64_G_65_G_66_G_67_G_68_**
NMS 5–12	5′-G_1_G_2_G_3_G_4_A_5_G_6_G_7_G_8_G_9_C_10_T_11_G_12_G_13_G_14_A_15_G_16_G_17_G_18_C_19_C_20_C*_21_**G_22_G_23_A_24_G_25_G_26_G_27_G_28_ G_29_C_30_T_31_G_32_G_33_G_34_C_35_C_36_G_37_G_38_G_39_G_40_A_41_C_42_C_43_C_44_G_45_G_46_G_47_A_48_G_49_G_50_G_51_G_52_T_53_C_54_G_55_G_56_ G_57_A_58_C_59_G_60_G_61_G_62_G_63_C_64_G_65_G_66_G_67_G_68_**
NMS 1–4	5′-**G_1_G_2_G_3_G_4_A_5_G_6_G_7_G_8_G_9_C_10_T_11_G_12_G_13_G_14_A_15_G_16_G_17_G_18_C_19_C_20_**C*_21_G_22_G_23_A_24_G_25_G_26_G_27_G_28_G_29_ C_30_T_31_G_32_G_33_G_34_C_35_C_36_G_37_G_38_G_39_G_40_A_41_C_42_C_43_C_44_G_45_G_46_G_47_A_48_G_49_G_50_G_51_G_52_T_53_C_54_G_55_G_56_ G_57_A_58_C_59_G_60_G_61_G_62_G_63_C_64_G_65_G_66_G_67_G_68_
Truncated 1–4	5′- **G_1_G_2_G_3_G_4_A_5_G_6_G_7_G_8_G_9_C_10_T_11_G_12_G_13_G_14_A_15_G_16_G_17_G_18_C_19_C_20_C_21_**
Truncated 1–4 + 7-nt	5′-**G_1_G_2_G_3_G_4_A_5_G_6_G_7_G_8_G_9_C_10_T_11_G_12_G_13_G_14_A_15_G_16_G_17_G_18_****C_19_C_20_C_21_G_22_G_23_A_24_G_25_**

Note: The sequence in bold is the region sandwiched between the two dsDNA handles. The complete DNA sequence along with the flanking regions used for the synthesis of the various constructs are given in Supplementary Table S1. The cytosine with an asterisk (C*_21_) is modified by an alkyne group for the attachment of an azide-modified dsDNA handle.

### Native mechanical segmentation targeting the 5^th^–12^th^ G-tracts (NMS5–12) in the hTERT promoter

Since a truncated hTERT sequence that contains 5–12 G-tracts has been characterized by mechanical unfolding approaches previously (18), we first applied native mechanical segmentation on the G-quadruplex contained in this section (the NMS5–12 construct, see Table 1) for a direct comparison between the two approaches. As discussed above, two dsDNA handles were introduced at the C21 position using the click chemistry coupling and at the 3′-end (position 68) by enzymatic ligation, respectively (see Materials and Methods for details). These two duplex handles were then attached to the two optically trapped polystyrene particles through digoxigenin-antibody and biotin-streptavidin affinity interactions (Figure 1). Force ramping experiments (18) were carried out by moving one of the optically trapped beads using a motorized mirror (20). During this process, the tension inside the mechanically targeted segment increased. When the tension was greater than the mechanical stability of a folded structure in the segment, the folded species unraveled. The sudden unraveling produced an unfolding transition with a rupture force (*F*_rupture_), which reflects the mechanical stability. The transition was also characterized by a change in contour length (Δ*L*), which corresponds to the size of the folded structure. By contrast, the region outside the segment enclosed by the dsDNA handles did not experience any tension, and therefore, structures in this mechanically isolated region remained intact.

**Figure 2.**
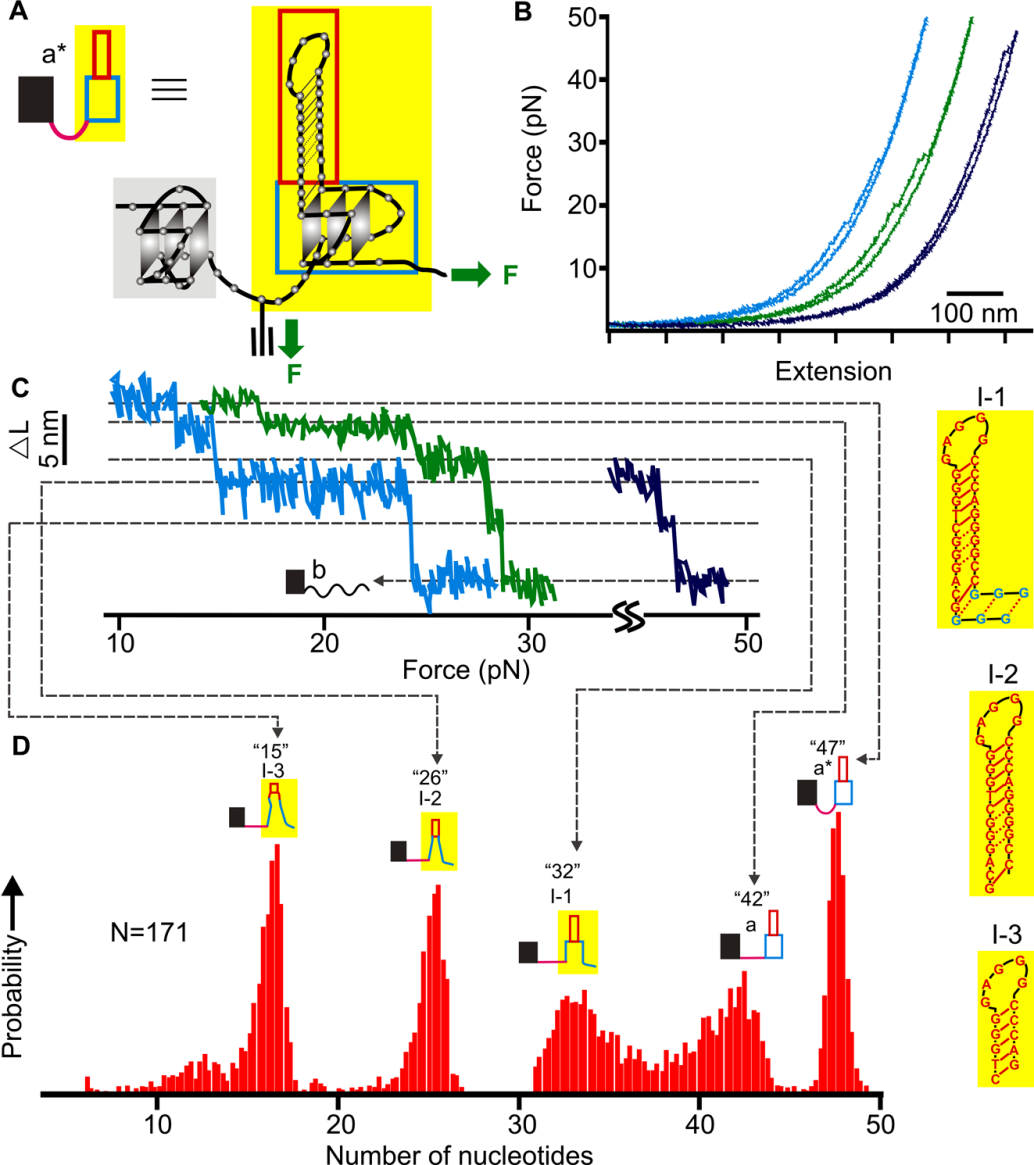
Mechanical unfolding of the NMS5–12 construct. (**A**) Schematic drawing of the native mechanical segmentation strategy. The green arrows depict attachments of two DNA handles that determine the direction of unfolding. (**B**) Representative *F-X* curves with different unfolding events from the same molecule. (**C**) Plot of change-in-contour-length (Δ*L*) versus force. Each plot was derived from a corresponding curve in B (see Supporting Information for details). (**D**) Probability of different populations identified from the PoDNano analysis (see Supporting Information). The number of nucleotides for each species is depicted on top of each structure diagram. Possible structures of three main intermediates are shown to the right. Solid and dotted red lines indicate Watson–Crick and Hoogsteen base pairings, respectively. See Supplementary Figures S6 and S7 for possible structures of all species.

Analyses on the unfolding patterns of this construct revealed multiple structures in the hTERT5–12 segment (Figure 2A). The change in contour length (Δ*L*) corresponding to each unfolding event was measured (see Supplementary Figure S6 for the overall Δ*L* histogram). To identify predominant species, we performed Population Deconvolution at Nanometer resolution (PoDNano, see Supporting Information) (21). First, we converted each force-extension curve to an *ΔL-F* plot, which allowed us to retrieve unfolding transitions with associated noise (standard error) in Δ*L*. Each Δ*L* was then expanded with a Gaussian kernel whose width was determined by the standard error. Combination of these Gaussian kernels then led us to identify predominant Δ*L* populations (Figure 2D) from 5000 resampling processes. Previously, such a procedure allowed deconvoluting Δ*L* populations as small as 0.47 nm in difference (21). Using Equation S1 described in Supporting Information, the number of nucleotides contained in predominate species can be calculated from Δ*L* measurements.

**Figure 3.**
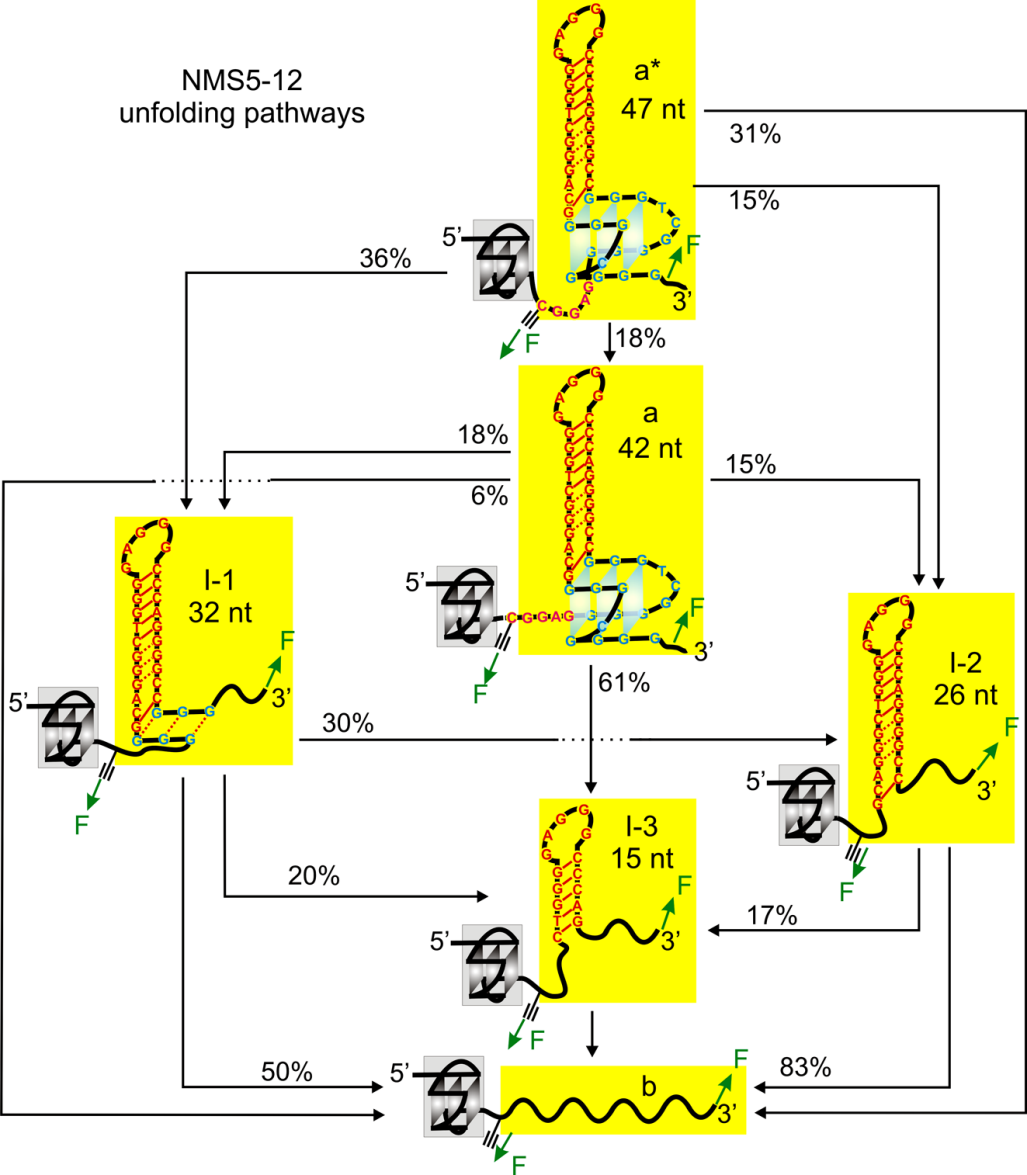
Unfolding pathways of the NMS5–12 construct based on the unfolding events observed in Figure 2. See Supplementary Figures S6 and S7 for detailed structures of each species. See Supplementary Table S2 for the number of molecules and traces used in the analyses. In each folded structure, solid and dotted red lines indicate Watson–Crick and Hoogsteen base pairings, respectively.

Since mechanical unfolding is a continuous process starting from the fully folded to fully unfolded states in a particular molecular segment, we were able to establish not only possible intermediates but also specific unfolding pathways by using this method (see Materials and Methods). As shown in Figure 2B–D, we identified five major folded populations containing 15, 26, 32, 42 and 47 nts during the unfolding of the NMS5–12. These species can convert into each other, forming a rather complex network of transition pathways. The 47-nt species matched the size of a G-quadruplex that employed 5^th^, 6^th^, 11^th^ and 12^th^ G-tracts with a 26-nt hairpin in the middle loop (Figure 1). The same structure has been observed previously (10,18). Evidence of the same G-quadruplex conformation also came from the CD spectrum of the alkyne modified hTERT 1–12 fragment, which displayed identical signatures with those in the wild type sequence (210 and 265 nm peaks, a 240 nm trough and a 290 nm shoulder, see Supplementary Figure S1) (10). Compared with the previous investigation on a truncated hTERT5–12 sequence (18), the intermediates with 15, 26 and 32 nucleotides were also observed here (see right insets of Figure 2D for possible structures). Close inspection on the unfolding pathways involving these three intermediates revealed the similarity between the native mechanical segmentation and the truncated mechanical unfolding. For example, in the truncated hTERT5–12 template, the 15-nt hairpin (similar to the I-3 in the right inset of Figure 2D) acts as a seed for the folding of the G-quadruplex in which the hairpin serves as one of the loops; the 26-nt (I-2) represents a longer hairpin in the same G-quadruplex loop; while the 32-nt (I-1) is likely an extended hairpin structure that contains three Hoogsteen guanine base pairs in the original G-quadruplex (G_32_G_33_G_34_/G_61_G_62_G_63_, see Table 1 for the sequence) (18). Based on these similarities, we assigned the 3 species (15, 26 and 32 nt, see I-1 to I-3 in Figures 2D and 3, and Supplementary Figure S6) in the NMS5–12 sequence as hairpin intermediates found in the truncated hTERT5–12 template. The 42-nt species is rather unique here. Since the entire structure should contain 47 nucleotides in total (48 nts if the last G (G68) in the 3′-end participates in the G-quadruplex, see Table 1), the observation of the 42-nt species suggests an intermediate with a similar size to the folded G-quadruplex in the NMS5–12. It is possible that the difference of the 5 nucleotides may stem from the inter-quadruplex region due to the higher-order interaction between the two G-quadruplexes (Figures 2A and 3, and Supplementary Figure S6) which was observed in the force range from 10–42 pN.

### Native mechanical segmentation targeting the 1^st^–4^th^ G-tracts (NMS1–4) in the hTERT promoter

To confirm that the 5-nt feature observed above is due to the disruption of the higher-order QQI under mechanical stress, we performed native mechanical segmentation on the G-quadruplex formed in the hTERT 1–4 G-tracts (the NMS1–4 construct). To mechanically target this 5′-end G-quadruplex, we designed a new strategy, Kinetic Enrichment of Ligation via Product Selection (KELPS, details see Materials and Methods and Supplementary Figure S3), to attach a dsDNA handle to the 5′-end of the same ODN 186 sequence used for the NMS5–12 construct preparation (Supplementary Table S1). In this method, we first ligated the alkyne modified ODN 186 with a single-stranded DNA (ODN 187.1, Supplementary Table S1) at the 5′-end using splint ligation (splint DNA: SSd003.2, see Supplementary Table S1) (9,19). Next, the splint DNA was replaced by another ssDNA (SSd003.3, see Supplementary Table S1) in which a toehold (22) section was introduced at the 5′-end. As a result of the hybridization with the second ssDNA, an adhesive overhang was introduced for the hTERT1–12 template, allowing ligation with the dsDNA handle by T4 DNA ligase. This synthesis exploited two kinetic features, a toehold strategy and a concentration based enhancement of the Watson–Crick hybridization reaction. The final covalent ligation with the dsDNA handle made this kinetic selection irreversible, achieving the enrichment of desired product. Since no purification is necessary, such a method is rather efficient in the modification of long DNA sequences, which are synthetically challenging. After attachment of the first dsDNA handle, click chemistry reaction (16) was then used to attach a second dsDNA handle to the C21 residue of the ODN186, accomplishing the NMS1–4 construct preparation.

**Figure 4.**
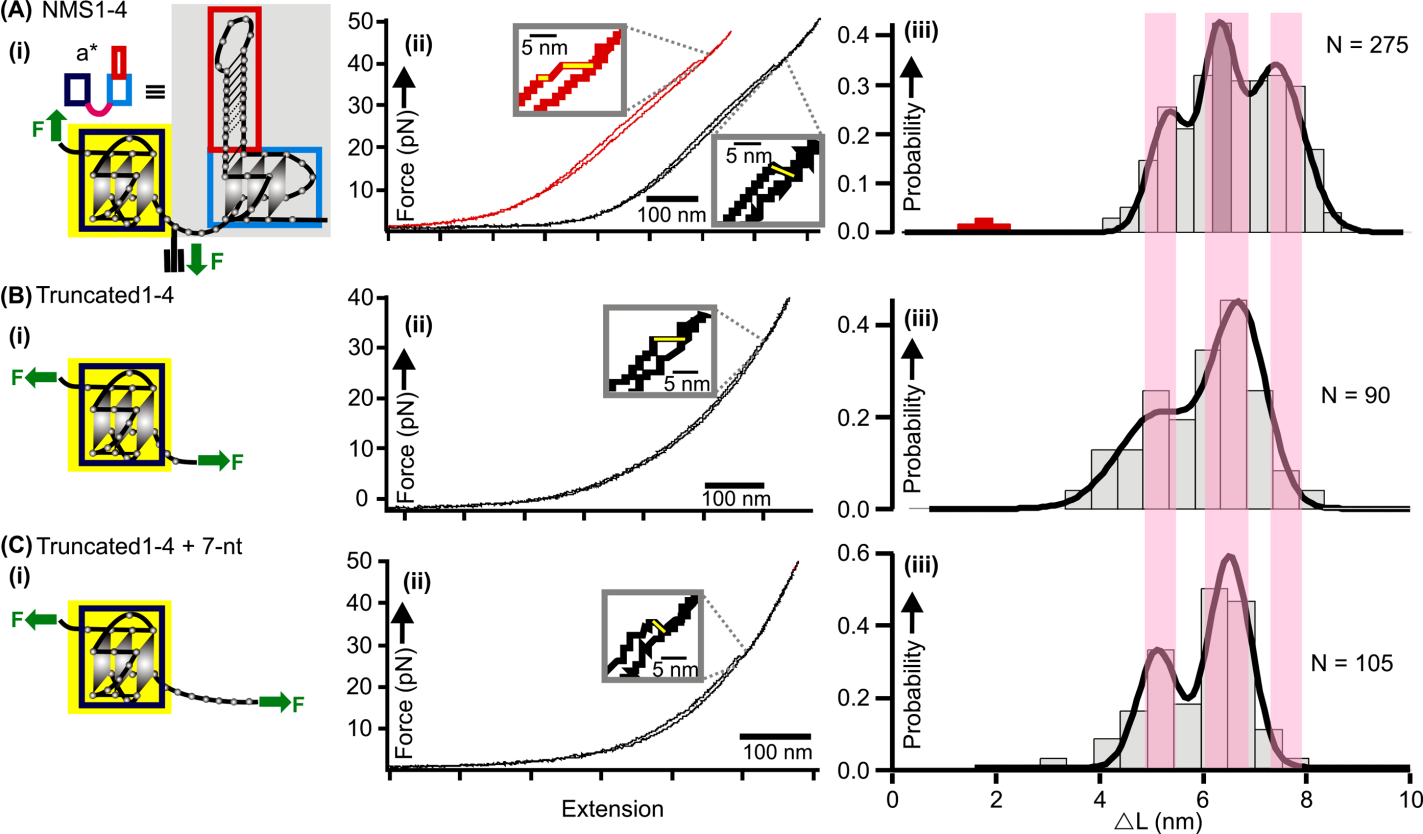
Mechanical unfolding of the NMS1–4 construct (**A**) and truncated1–4 constructs (**B** and **C**). (i) Schematic diagram of the mechanical unfolding for each construct. The green arrows depict the attachment of DNA handles that determine the direction of unfolding. (ii) Typical *F-X* traces observed for different constructs. (iii) Δ*L* histograms of the unfolding transitions observed in respective constructs. Notice the ≈1.2 nm population (red) in (A-iii) corresponds to the first unfolding feature in the red trace of (A-ii). See Supplementary Table S2 for the number of molecules and traces used in the analyses.

The results of the force ramping experiments on the NMS1–4 construct were shown in Figure 4. Although most *F-X* curves depicted only one sudden rupture event, some traces exhibited two-step unfolding events (see red trace in Figure 4A). Statistical analyses on the Δ*L* histogram (Figure 4A-iii) showed three significant populations centered at 4.9 nm, 6.3 nm and 7.5 nm, respectively. Calculation revealed these populations contained 14, 18 and 21 nucleotides, respectively (see Equation S1 in Supporting Information). While the 18-nt species has the number of nucleotides consistent with those involved in the fully folded G-quadruplex in the 1–4 G-tracts, the 14-nt species is likely an intermediate that may assume a G-triplex conformation (23,24). In comparison, Δ*L* histogram of the structures formed in the truncated sequence, 5′-GGG GAG GGG CTG GGA GGG CCC (or truncated1–4), also showed these two populations (Figure 4B-iii), therefore validating the assignment of their respective structures.

### High-order interaction between the two neighboring G-quadruplexes

The population with Δ*L* = 7.5 nm in the NMS1–4 construct is rather unique. This population did not appear in the truncated1–4 sequence, suggesting the contribution of the 3′-end G-quadruplex (hTERT 5–12 G-tracts) to the presence of this feature. The Δ*L* difference between this species and that of the 5′-end G-quadruplex (7.5 – 6.3 = 1.2 nm) is consistent with the length of the inter-quadruplex region (3 nucleotides, see Equation S1 for calculation) between the 5′-end G-quadruplex (hTERT 1–4 G-tracts) and the alkyne-modified C21 residue. Therefore, the Δ*L* = 7.5 nm population could stem from the simultaneous unfolding of the tertiary QQI and the 5′-end G-quadruplex in the NMS1–4 construct. Occasionally, the unfolding of these two structural elements was sequential (see red data in Figure 4A(ii) and (iii)), supporting this assignment. Further support came from the unfolding of a control construct, 5′-GGG GAG GGG CTG GGA GGG CCC GGA G, which contained the complete inter-quadruplex sequence at its 3′-end (underlined, see ‘Truncated1–4 + 7-nt’ in Table 1). Similar to the truncated1–4 construct, we again found the 7.5 nm feature was missing (compare Figure 4B-iii and C-iii). This indicated that the inter-quadruplex sequence itself did not fold into any structure without the presence of the G-quadruplex at the 3′-end of the hTERT1–12 fragment.

It is likely that the 7-nt bridging segment may assume a looped-out conformation when there is high-order interaction, such as stacking (25), between the two hTERT G-quadruplexes. Unfolding of either the 5′-end or the 3′-end G-quadruplex during the native mechanical segmentation involves a common residue, C21, in the inter-quadruplex region. Under mechanical tension, the looped-out bridge region will release seven nucleotides as tertiary QQI becomes disrupted. Given the stacking interaction between the terminal G-quartets of the two G-quadruplexes, the NMS1–4 and NMS5–12 constructs should produce rupture features of 3 nts and 5 nts (the C21 residue is counted in both cases) from the 7-nt inter-quadruplex region when the 5′-end (1–4 G-tracts) and the 3′-end (5–12 G-tracts) G-quadruplexes are unfolded, respectively (see Equation S1 in Supporting Information for calculation). Actual observation of these two expected features (the 1.2 nm species in Figure 4A [the first transition in the red trace] and the 1.7 nm species in Figure 2C,D [transition from a* to a]), therefore, provided a strong support for this type of stacking interaction between the two hTERT G-quadruplexes.

Consistent with the tertiary interactions between the two G-quadruplexes, we observed many cooperative unfolding events in which both the high-order interaction and the G-quadruplex in the mechanically targeted segment unfolded simultaneously. For example, in the 5′-end G-quadruplex (NMS1–4), the cooperative unfolding constituted 30% of all events (Figure 4A) whereas it was 31% in the 3′-end G-quadruplex (NMS5–12, Figure 3). Previously, these cooperative unfolding events have been ascribed to the high-order interactions in proteins (26).

The assignment of the tertiary interaction in the NMS1–4 construct allowed us to calculate the change in free energy of the higher order structure (Δ*G*_tertiary_) by converting the unfolding work of the tertiary interaction using the Jarzynski non-equilibrium equality equation (Equation S2 in Supporting Information) (17,27). Since change in free energy is a state function which does not depend on specific unfolding pathways, unfolding work of either the NMS1–4 or NMS5–12 construct can be used to retrieve Δ*G*_tertiary_ (17). The NMS5–12, however, contained unfolding patterns too complex to deconvolute accurately. Therefore, we proceeded to obtain Δ*G*_tertiary_ from the unfolding pattern of the NMS1–4 construct. We first calculated the change in free energy of unfolding G-quadruplex alone (Δ*G*_quadruplex_, the 6.3 nm population in Figure 4A) and that of unfolding G-quadruplex and tertiary structure together (Δ*G*_quadruplex+tertiary_, the 7.5 nm population in Figure 4A). Due to the state function nature, the Δ*G*_tertiary_ was determined as the difference between these two values, which exhibited a value of 2.0 ± 0.4 kcal/mol (see Supplementary Figure S5 and Supporting Information). Compared with the 6 kcal/mol stabilization energy between the 26-nt DNA hairpin and the main G-quadruplex in the truncated hTERT5–12 sequence (mainly H-bonding and van der Waals force, see reference (18)), the tertiary interaction observed here between the two G-quadruplexes (mainly π–π interactions) is significantly weaker. This result is consistent with recent finding that loop–loop interaction mediated by H-bonding and van der Waals force is stronger than the π–π interactions in human telomeric G-quadruplex (28).

### Unfolding pathways of the species in the full hTERT1–12 sequence

**Figure 5.**
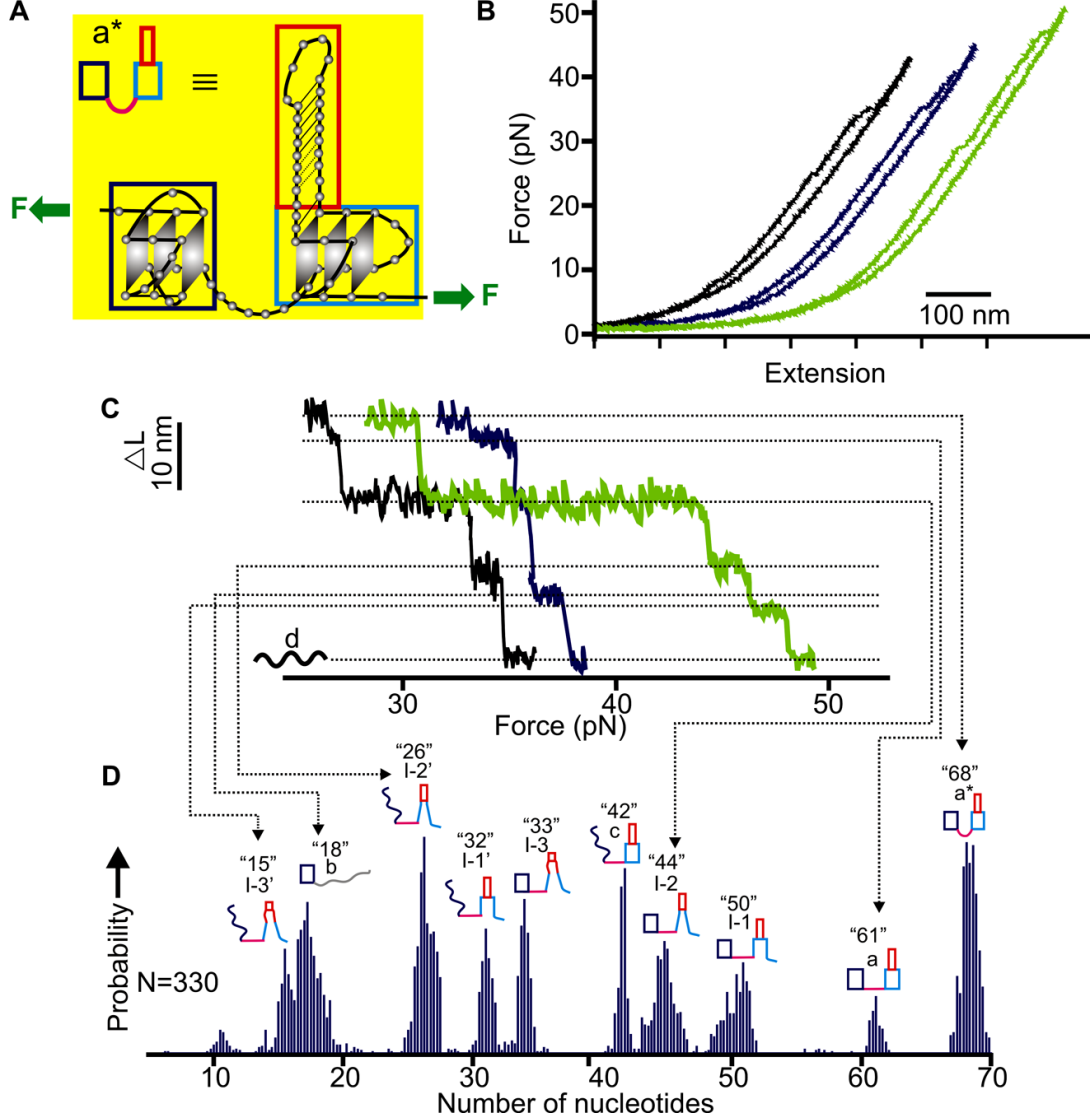
Mechanical unfolding of the hTERT1–12 sequence. (**A**) Schematic drawing of the mechanical unfolding strategy. The green arrows depict attachments of two DNA handles that determine the direction of unfolding. (**B**) Representative *F-X* curves with different unfolding events from the same molecule. (**C**) Plot of change-in-contour-length (Δ*L*) versus force from the traces shown in (B). For clarity, only major transitions are shown. See Supplementary Figure S9 for all other transitions. (**D**) Probability of all unfolding populations obtained from the PoDNano analysis (see Supporting Information). Number of nucleotides for each species is depicted on top of each structural diagram. See Supplementary Figures S6 and S7 for detailed structures and Supplementary Table S2 for the number of molecules and traces used in the analyses.

If the stacking interaction indeed exists between the two G-quadruplexes, unfolding of the entire hTERT 1–12 sequence should reveal this interaction as well. To test this prediction, a DNA construct was synthesized by sandwiching the full hTERT 1–12 G-tracts sequence (68 nts, see Table 1) between two dsDNA handles (see Materials and Methods and Supplementary Figure S2 for preparation). The DNA construct was then subject to the force ramping procedures, which produced multiple unfolding events in most *F-X* traces (Figure 5). By measuring Δ*L*, the intermediates that lead to specific unfolding events can be analyzed. However, there is uncertainty in the assignment of these intermediates as molecules can be broken prematurely during stretching or they may not be fully refolded during the incubation before subsequent force-ramping cycles. To account for these uncertainties, only the molecules that showed both fully folded and fully unfolded traces in any of the repetitive *F-X* curves were used for analysis (see Supplementary Figure S6 for overall Δ*L* histograms).

Except for a few cases (see green trace in Figure 5B and C), majority of the curves showed individual transitions with Δ*L* = 2.5 nm. This number is equivalent to 7 nucleotides (see Equation S1 for calculation), which is consistent with those contained in the inter-quadruplex region. Such a result is in agreement with the disruption of the stacking interaction between the two G-quadruplexes. It is notable that the high-order interaction almost always appeared as the first event during the stretching of the DNA construct in a wider force range from 10–50 pN. In the case of the green trace in Figure 5B and C, we observed an 8 nm Δ*L* during the first transition event. Such a transition suggests simultaneous disruption of the QQI and the unfolding of the G-quadruplex from the hTERT 1–4 G-tracts (the expected Δ*L* is 8.4 nm, see Equation S1 for calculation). This result implied that the G-quadruplex from the 5′-end G-quadruplex (hTERT 1–4 G-tracts) was weaker than that formed in the 5–12 G-tracts.

**Figure 6.**
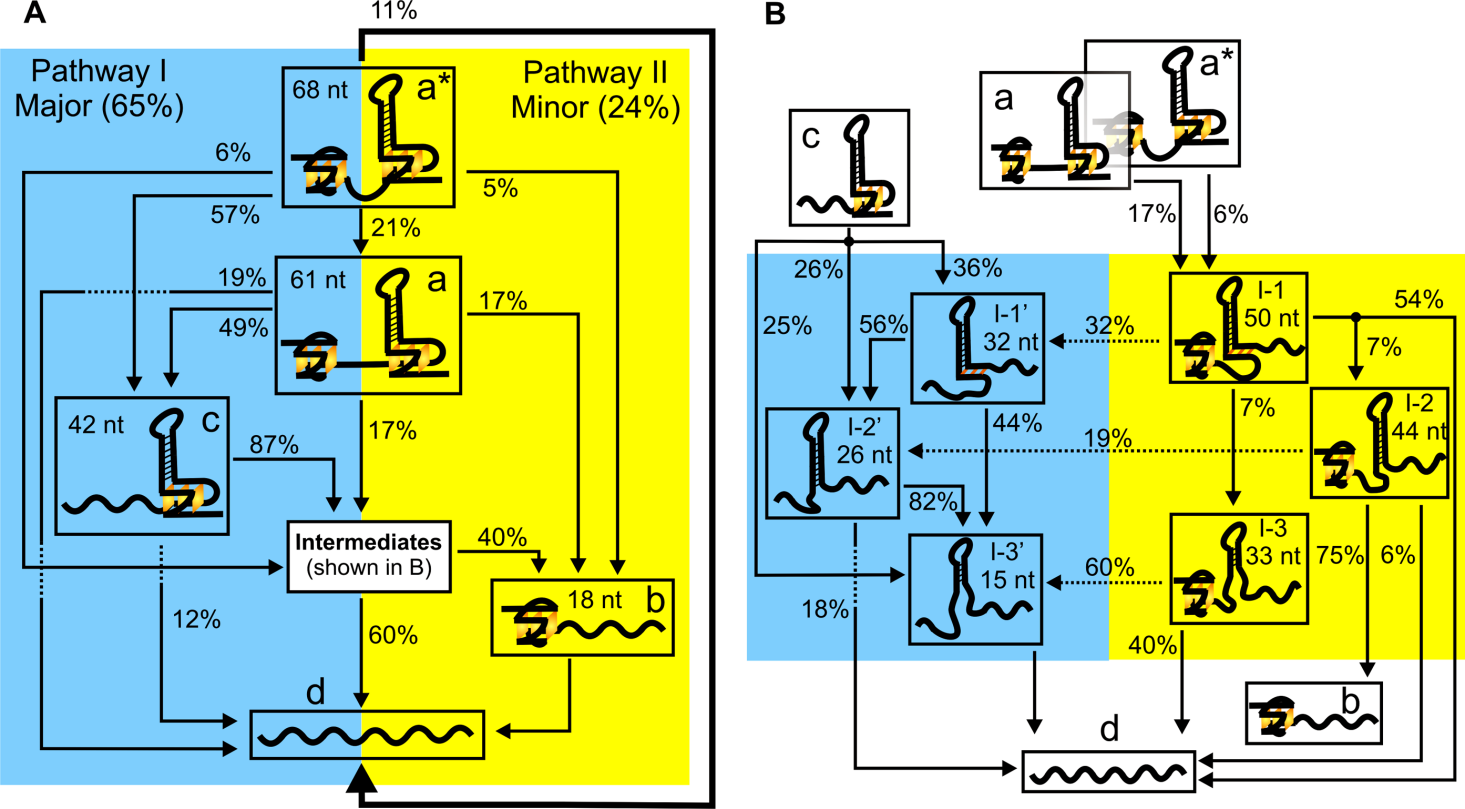
Unfolding pathways of the hTERT1–12 construct based on the unfolding events observed in Figure 5. (**A**) Schematic drawing of the unfolding through the 5′-end G-quadruplex (65%, blue) or the 3′-end G-quadruplex (24%, yellow). (**B**) Unfolding pathways involving various intermediates. See Supplementary Table S2 for the number of molecules and traces used in the analyses.

Based on the size (Δ*L*) of the intermediates and the order of occurrence of these rupture events (see Materials and Methods and Supporting Information), next, we compiled the unfolding pathways of completely folded structures in the hTERT1–12 construct (Figure 6). All the transitions observed can be ascribed to the unfolding through either the 5′-end (hTERT 1–4 G-tracts) or the 3′-end (hTERT 5–12 G-tracts) G-quadruplex. During the unfolding through the 3′-end G-quadruplex, we observed three types of intermediates, I-3′/I-3, I-2′/I-2 and I-1′/I-1 (Figure 6B and Supplementary Figure S8). The sizes of these species were similar to those found in the native mechanical segmentation of the NMS5–12 construct (Figures 2 and 3) as well as those in the truncated hTERT5–12 sequence (18), which validated our pathway assignments (Figure 6A). Detailed analysis revealed that 11% of all unfolding events were direct unfolding from fully folded structure (a*) to fully unfolded population (d). Such cooperative unfolding confirmed the presence of the higher order interaction between the two hTERT G-quadruplexes (26). For the rest of the molecules, majority (65%) preferred to unfold through the G-quadruplex formed in the 1–4 G-tracts, followed by the unfolding of the hTERT 5–12 G-quadruplex. In contrast, only 24% unfolded with a reverse order (unfolding of the 3′-end G-quadruplex followed by the 5′-end G-quadruplex). This is likely due to the stronger mechanical stability of the 3′-end G-quadruplex, a result consistent with the above observation in which it was easier to unfold the higher order interaction and the 5′-end G-quadruplex together, rather than that with the 3′-end G-quadruplex together. The stronger mechanical stability in the 3′-end G-quadruplex was likely contributed from the tertiary interaction between the 26-nt hairpin loop and the G-quadruplex in the hTERT 5–12 G-tracts, which provided an additional stability of 6 kcal/mole (18). The hierarchical unfolding of the two G-quadruplex structures in the complex DNA structure confirmed the finding (29) that due to limited long range interactions between different structures in nucleic acids, the folding/unfolding pathways in RNA/DNA structures are hierarchical, which is markedly different from the cooperative transition pathways exhibited in proteins.

It is noteworthy that the unfolding pattern in the hTERT1–12 sequence did not support the reported model (30) that three stacking G-quadruplexes are formed in this DNA fragment. Calculations according to this model revealed that unfolding transitions with Δ*L* of 6.2 nm (the 5′-end G-quadruplex), 8.6 nm (the middle G-quadruplex) and 7.0 nm (the 3′-end G-quadruplex) should be observed for the three stacking G-quadruplexes counting from the 5′ end, respectively (see Supplementary Table S3). Although the 6.2 and 7.0 nm species were present, the 8.6 nm population in the full hTERT1–12 sequence decreased (see arrows in Supplementary Figure S6), implying that formation of three G-quadruplexes is not likely in the hTERT1–12 promoter. In addition, if the stacking model is correct, it is expected to form 8.6 nm and 7.0 nm G-quadruplex species in the NMS5–12 construct. Consistent with the observation in the hTERT1–12, however, the 8.6 nm G-quadruplex was absent in the NMS5–12 construct (Supplementary Figure S6), which again indicates that stacking of the 3 consecutive G-quadruplexes is not likely.

## CONCLUSION

In this work, we developed a new mechanical unfolding strategy to analyze higher order interactions in biomacromolecules. Using click chemistry, we were able to mechanically target a desired domain in a biomolecule while leaving the rest of the structure free of mechanical stress. Similar to surgical operations, such a strategy allowed revealing the property of different structural domains in a native environment with a highly precise manner. Our results clearly indicated the existence of tertiary interaction between the two G-quadruplexes formed in the hTERT promoter fragment. The tertiary interaction occurs through the π–π interactions between the two G-quartets at the inter-quadruplex region and provides 2 kcal/mol in stabilization energy. In addition, we found the unfolding pathways of a full structure can be reconstituted from those of individual G-quadruplex units, confirming the hierarchical nature of nucleic acid structures. Analogous to the exploded representation in the mechanical drawing, by employing a generic approach of click chemistry, we anticipate this strategy can provide an exploded mechano-analytical perspective (XMAP) not only to nucleic acid tertiary structures but also to long range interactions in proteins.

## Supplementary Material

SUPPLEMENTARY DATA
