# Supplementary material for: Exploded view of higher order G-quadruplex structures through click-chemistry assisted single-molecule mechanical unfolding
Source: Nucleic Acids Res. 2015 Nov 30;44(1):45–55. doi: 10.1093/nar/gkv1326 (PMC4705664; doi:10.1093/nar/gkv1326)
Supplement: SUPPLEMENTARY DATA [file supp_44_1_45__index.html]

Exploded view of higher order G-quadruplex structures through click-chemistry assisted single-molecule mechanical unfolding — Exploded view of higher order G-quadruplex structures through click-chemistry assisted single-molecule mechanical unfolding — SUPPLEMENTARY DATA 

# Exploded view of higher order G-quadruplex structures through click-chemistry assisted single-molecule mechanical unfolding

## SUPPLEMENTARY DATA

- SUPPLEMENTARY DATA
