# Supplementary material for: Exploded view of higher order G-quadruplex structures through click-chemistry assisted single-molecule mechanical unfolding
Source: Nucleic Acids Res. 2015 Nov 30;44(1):45–55. doi: 10.1093/nar/gkv1326 (PMC4705664; doi:10.1093/nar/gkv1326)
Supplement: SUPPLEMENTARY DATA [file supp_gkv1326_nar-02611-f-2015-File002.docx]

**Exploded View of Higher Order G-quadruplex Structures through Click-Chemistry Assisted Single-Molecule Mechanical Unfolding**

Sangeetha Selvam, Zhongbo Yu and Hanbin Mao*

Department of Chemistry and Biochemistry, Kent State University, Kent, Ohio, 44242, USA

*Corresponding author: HM, hmao@kent.edu (330-672-9380)

**Estimation of the number of nucleotides involved in a particular structure**

The number of nucleotides (*N*) involved in a particular population can be calculated using the following equation ([1](#_ENREF_1),[2](#_ENREF_2)),

$\Delta L=\left( N*L_{sn} \right)-x$ ……..Eqn (S1)

where *L*_sn_ is the contour length per nucleotide in single-stranded DNA (0.4 nm)([3-5](#_ENREF_3)) and *x* is the end-to-end distance of a folded structure. For various structures, *x* is 1 nm for G-quadruplex (from 5′- to 3′-end) ([6](#_ENREF_6)), 1.2 nm for the G-G Hoogsteen base pairs ([6](#_ENREF_6)), 1.8 nm for the Watson-Crick base pairs ([7](#_ENREF_7)), 1.5 nm for G-triplex ([8](#_ENREF_8)), or 0.6 (±0.4 nm) for quartet-to-quartet distance, which is measured from the NMR structure of the hTERT 1-4 G-quadruplex (PDB 2KZD) ([6](#_ENREF_6)).

From the equation S1, the number of nucleotides involved in the Δ*L* = 2.3 nm unfolding feature in the hTERT1-12 construct (Figure 5B&C) was estimated to be ~7 nucleotides with *x =* 0.6 (±0.4) nm ([6](#_ENREF_6)) and *L*_sn_ = 0.4 nm.

For the NMS1-4 construct, three Δ*L* populations were observed at 4.9 nm, 6.3 nm, and 7.5 nm (Figure 4A), respectively. The 4.9 nm population contained ~ 16 nts (*L*_sn_ = 0.4 nm and *x* =1.5 (±0.1) nm). This was close to that involved in the G-triplex intermediate (14 nts were expected to form a triplex, 5′-G_1_G_2_G_3_G_4_A_5_G_6_G_7_G_8_G_9_C_10_T_11_G_12_G_13_G_14_, inside the NMS1-4 construct, see Table 1). The 6.3 nm population showed that 18 nts were involved in the structure (*L*_sn_ = 0.4 nm and *x* = 1 nm). The 7.5 nm population corresponded to 22 nts (*L*_sn_ = 0.4 nm and *x*= 1.26 nm [this is equivalent to the 1 nm end-to-end distance of a G-quadruplex and the estimated 0.26 nm end-to-end distance estimated from the 3 nts out of 7 nts in the inter-quadruplex stacking region (0.6 nm * 3/7 = 0.26 nm)]). In comparison, the possible structure formed the targeted region consists of 21 nts (see bold region of the NMS1-4 in Table 1).

With *x* as 0.26 nm and 0.34 nm for the NMS1-4 and the NMS5-12 constructs, respectively, the number of nucleotides involved in the 1.2 nm (Figure 4A, the difference between 7.5 and 6.3 nm species) and the 1.7 nm species (Figure 2C&D: transition from a* to a) were 3 nts and 5 nts, respectively (*L*_sn_ = 0.4 nm ([3-5](#_ENREF_3))).

In the hTERT1-12 construct, the number of nucleotides involved in the simultaneous unfolding of the 5′-end G-quadruplex and the quadruplex-quadruplex interaction is 25 nts (18 nts from G-quadruplex with 7 nts from the inter-quadruplex region). The expected Δ*L* for this transition was calculated to be 8.4 nm with *L*_sn_ = 0.4 nm ([3-5](#_ENREF_3)) and *x* = 1.6 nm (summation of *x* = 1 nm for G-quadruplex and *x* = 0.6 nm ([6](#_ENREF_6)) for the 7-nt stacking region between the two quadruplexes).

**Deconvolution of Δ*L* populations**

To calculate the change in the unfolding free energy of a specific population (see above), it is necessary to deconvolute unfolding events corresponding to the population. First, the Δ*L* histogram (Figure 4A-iii) was fit with a three-peak Gaussian centered at 4.9 nm, 6.3 nm, and 7.5 nm, respectively. Random deconvolution strategy ([9](#_ENREF_9)) was used to assign the events in the overlapping region to one of the two neighboring populations. The ratio of the events between the two populations in each bin of the overlapping region was determined by the Gaussian fitting described above. From the deconvoluted events, work histogram was constructed for the 6.3 nm and the 7.5 nm species separately (see Figure S5).

**Calculation of change in free energy of unfolding (Δ*G*_unfold_)**

Δ*G*_unfold_ was calculated using Jarzynski non-equilibrium equality equation ([3](#_ENREF_3),[10](#_ENREF_10)),

$\Delta G_{unfold}= -k_{B}Tln\sum_{i=1}^{N} \frac{1}{N}exp\left( -\frac{W_{i}}{k_{B}T} \right)$ ……..Eqn (S2)

where *N* is the number of features observed in the experiment and *W* is the work done to unfold a G-quadruplex,

$W=\sum_{i=1}^{N_{s}} F_{i}\Delta x_{i}$

here *F* is the force corresponding to the unfolding event and Δ*x* is the change in extension during the unfolding of a structure.

**PodNano (Population Deconvolution at Nanometer resolution) analysis**

First, an Δ*L*-*F* plot was generated from each *F*-*X* curve in the full force range (1 - 60 pN) using the following equation S3 ([3](#_ENREF_3)).

$\Delta L= \Delta x\left( 1-\frac{1}{2}\left( \frac{k_{B}T}{FP} \right)^{\frac{1}{2}}+\frac{F}{S} \right)^{-1}$……. Eqn (S3)

where Δ*L* is the change in contour length, Δ*x* is the change in end-to-end distance, *F* is the force at which the sudden unfolding event occurs, *P* is the persistent length of dsDNA (50.8 nm) ([11](#_ENREF_11)), *S* is the stretch modulus of dsDNA (1243 nm) ([11](#_ENREF_11)), *k*_B_ is the Boltzmann constant, and *T* is absolute temperature.

From each Δ*L*-*F* plot, a Gaussian kernel density (Equation S4) was used to estimate the probability-density distribution (*p*) of each Δ*L* transition ([3](#_ENREF_3),[12](#_ENREF_12)).

$p= \frac{1}{\sigma\sqrt{2\pi}}exp\left( -\frac{\left( x-\Delta L \right)^{2}}{{2\sigma}^{2}} \right)$…….Eqn (S4)

where *x* refers to the range Δ*L* ± 3*σ*, *σ* is the average standard error from the immediate regions flanking a specific transition. After obtaining the kernel density distribution, bootstrapping was performed to identify 3 predominant species from each of the 5000 resampling processes. These predominant species constituted a histogram (Figures 2D and 5D), from which the major Δ*L* species were then identified during the unfolding of the structures formed in a specific construct.

Table S1. List of DNA sequences used in this study

| **Oligo Name** | **Sequence (5′→3′)** | **DNA construct name** |
| --- | --- | --- |
| ODN 185  (134 nts) | CTA GAC GGT GTG AAA TAC CGC ACA GAT GCG TTT TTG GGG AGG GGC TGG GAG GGC CCG GAG GGG GCT GGG CCG GGG ACC CGG GAG GGG TCG GGA CGG GGC GGG GTT TTT GCC AGC AAG ACG TAG CCC AGC GCG TC | hTERT1-12 (Sequence of interest (SOI) with flanking regions) |
| SSd004.2  (87 nts) | CTA GAC GGT GTG AAA TAC CGC ACA GAT GCG TTT TTG GGG AGG GGC TGG GAG GGC CCTT TTT GCC AGC AAG ACG TAG CCC AGC GCG TC | Truncated1-4 (SOI with flanking regions) |
| SSd004.3  (91 nts) | CTA GAC GGT GTG AAA TAC CGC ACA GAT GCG TTT TTG GGG AGG GGC TGG GAG GGC CCG GAG TTT TTG CCA GCA AGA CGT AGC CCA GCG CGT C | Truncated1-4 + 7nt (SOI with flanking regions) |
| ODN 186  (97 nts) | G GGG AGG GGC TGG GAG GGC CC*G GAG GGG GCT GGG CCG GGG ACC CGG GAG GGG TCG GGA CGG GGC GGG GTT TGC CAG CAA GAC GTA GCC CAG CGC GTC | NMS1-4 (SOI with flanking regions) |
| ODN 186  (97 nts) | G GGG AGG GGC TGG GAG GGC CC*G GAG GGG GCT GGG CCG GGG ACC CGG GAG GGG TCG GGA CGG GGC GGG GTT TGC CAG CAA GAC GTA GCC CAG CGC GTC | NMS5-12 (SOI with flanking regions) |
| ODN 187.1  (31 nts) | GGC CGC ATC TGT GCG GTA TTT CAC ACC GTT T | NMS1-4 (used in the KELPS strategy) |
| SSd003.2  (57 nts) | CCCAGCCCCCTCCGGGCCCTCCCAGCCCCTCCCCAAACGGTGTGAAATACCGCACAG | NMS1-4 (used in the KELPS strategy) |
| SSd003.3  (24 nts) | CGGTGTGAAATACCGCACAGATGC | NMS1-4 (used in the KELPS strategy) |

Note: The underlined sequence is the sequence of interest in respective constructs.

Table S2. Data collected on each DNA construct for single-molecule analysis

| **DNA construct** | **Number of features (N)** | **Number of curves (C)** | **Number of molecules (M)** |
| --- | --- | --- | --- |
| NMS5-12 | 171 | 150 | 18 |
| NMS1-4 | 275 | 271 | 26 |
| Truncated1-4 | 90 | 90 | 37 |
| Truncated1-4 + 7 nt | 105 | 105 | 26 |
| hTERT1-12 | 330 | 173 | 25 |

Table S3. Expected Δ*L* for each G-quadruplex in the hTERT1-12 as proposed in the three-G-quadruplex-stacking model ([13](#_ENREF_13))

| **Sequence in the hTERT1-12** | **Nucleotides involved in G-quadruplex** | **Expected Δ*L*** |
| --- | --- | --- |
| **G_1_G_2_G_3_G_4_A_5_G_6_G_7_G_8_G_9_C_10_T_11_G_12_G_13_G_14_A_15_G_16_G_17_G_18_** | 18 nt | 6.2 nm |
| **G_25_G_26_G_27_G_28_G_29_C_30_T_31_G_32_G_33_G_34_C_35_C_36_G_37_G_38_G_39_G_40_A_41_C_42_C_43_C_44_G_45_G_46_G_47_A_48_** | 24 nt | 8.6 nm |
| **G_49_G_50_G_51_G_52_T_53_C_54_G_55_G_56_G_57_A_58_C_59_G_60_G_61_G_62_G_63_C_64_G_65_G_66_G_67_G_68_** | 20 nt | 7.0 nm |


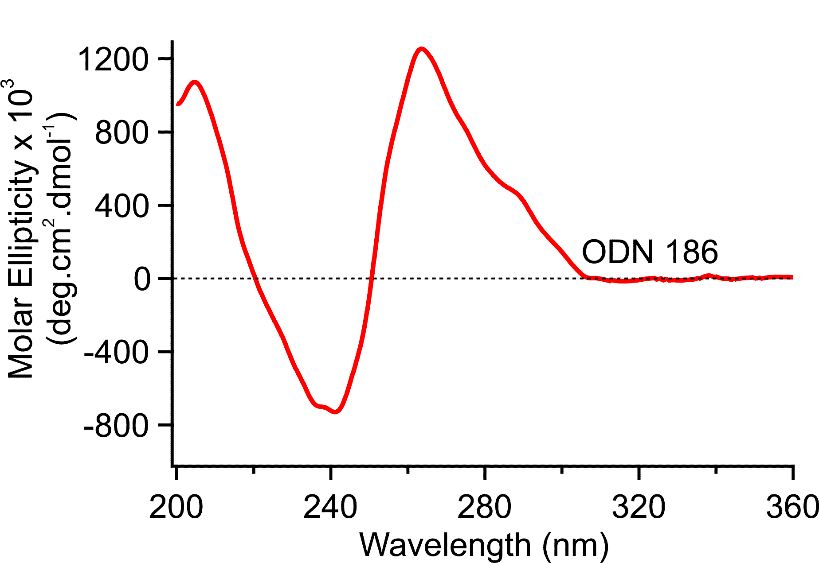


*Figure S1. Circular dichroic spectrum of the oligonucleotide ODN 186 (see Table S1 for sequence). Note this spectrum is identical with that of the wild type sequence without alkyne modification (see Figure 4A in reference (*[*14*](#_ENREF_14)*)).*


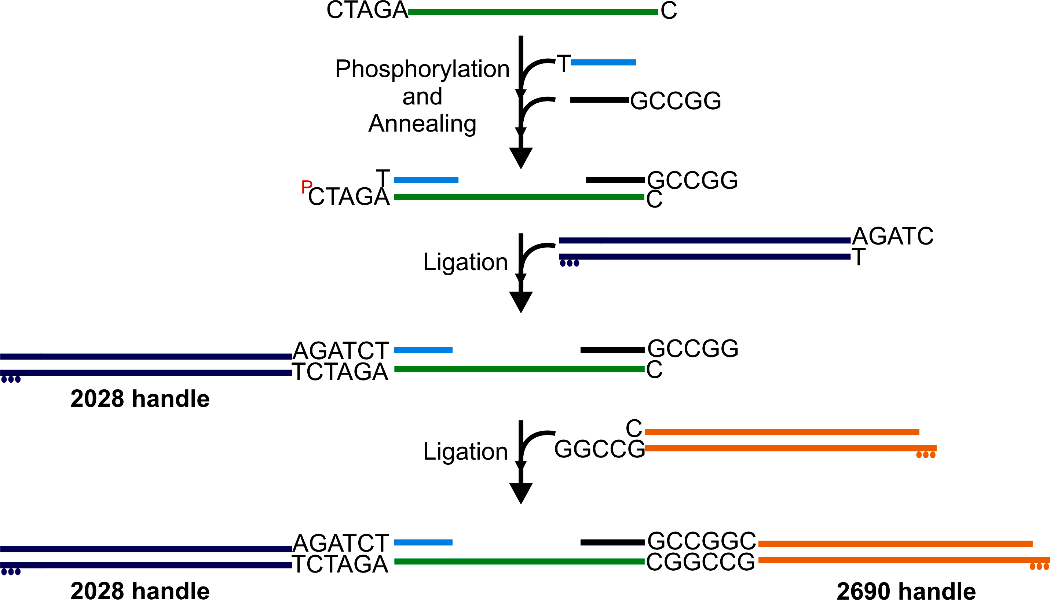


*Figure S2. Synthetic strategy of the hTERT1-12, the truncated1-4, and the truncated1-4 + 7-nt constructs. The green oligo represents the sequence of interest (SOI) flanked by the two 26-bp DNA regions, which are depicted by cyan and black colors at both ends, respectively.*


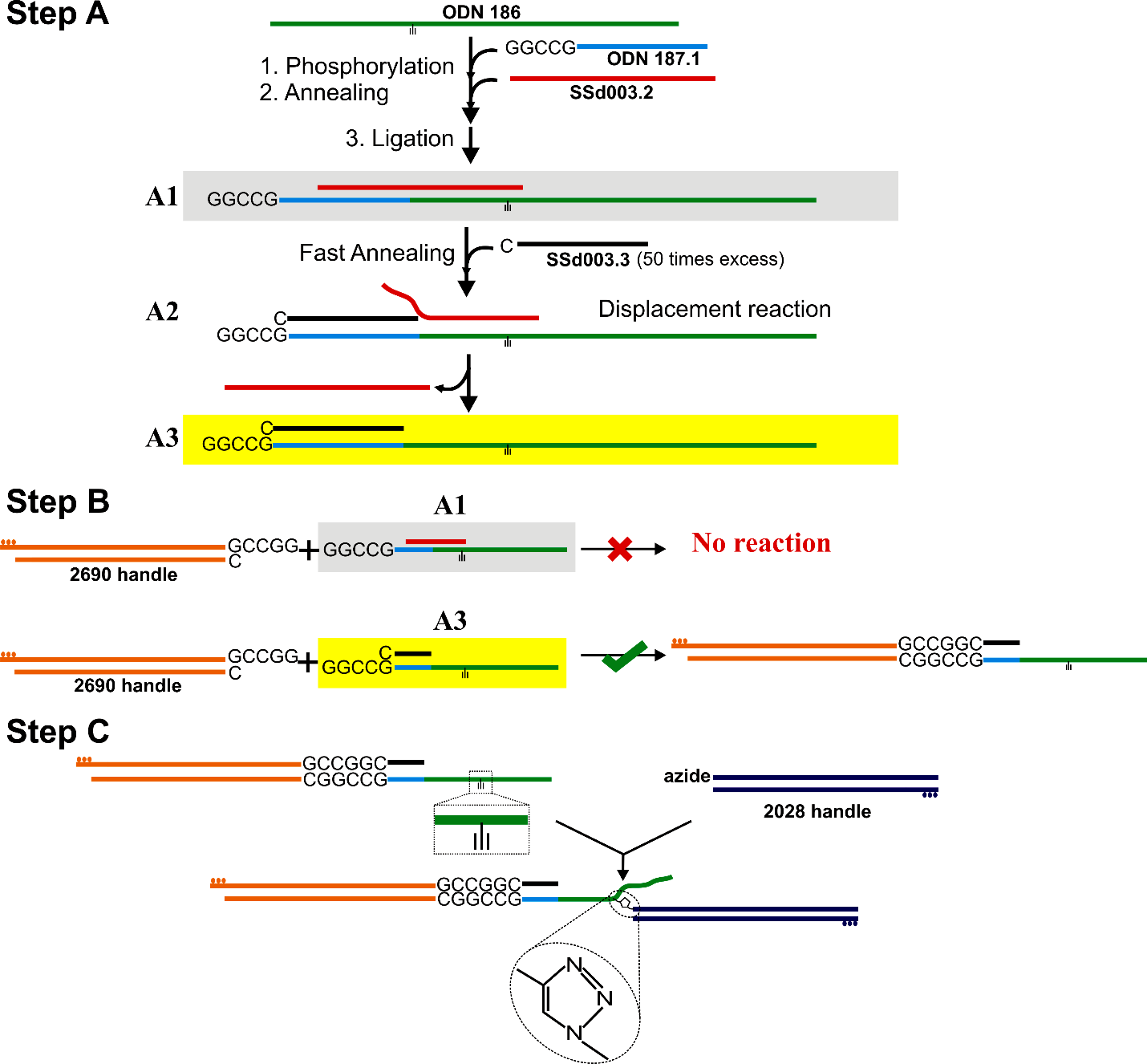


*Figure S3. Synthetic strategy for the Kinetic Enrichment of Ligation via Product Selection (KELPS) used for the synthesis of construct NMS1-4. For the preparation of NMS 5-12 construct, the synthetic strategy involves the steps B and C as shown above. See Materials and Methods for detailed description.*

*
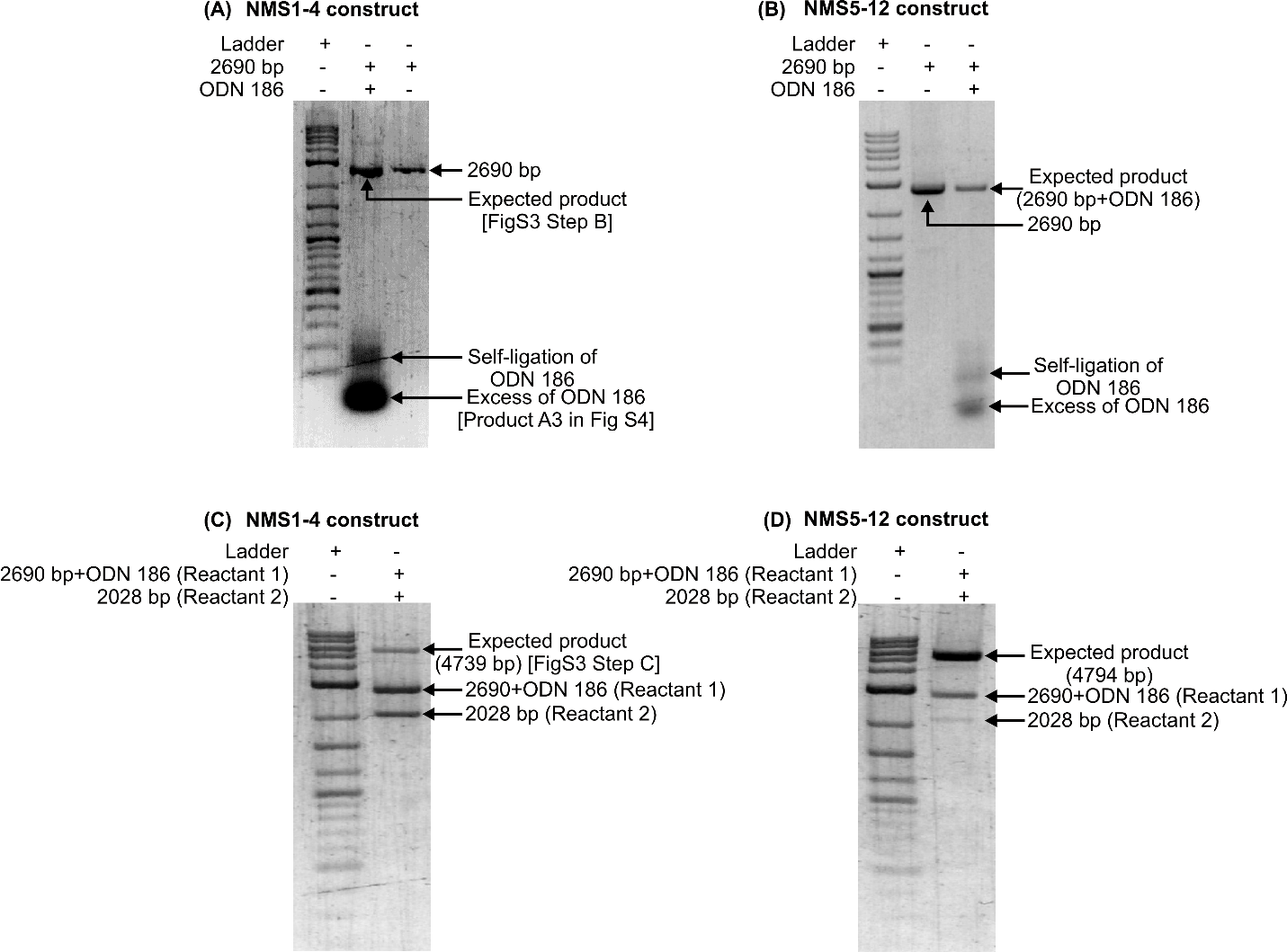
*

*Figure S4. Gel images of the ligation of the SOI sequences bearing alkyne-modified cytosines with the 2690 bp dsDNA handles for the NMS1-4 (A) and NMS5-12 (B) constructs, respectively. (C and D) Gel images of the click reaction of the ligated products from A and B with the 2028 bp dsDNA handles bearing azide modifications. From these gels, it is clear that click reaction was rather clean without side products. The efficiencies of the click reaction are 22% and 57% for the NMS1-4 and NMS5-12 constructs respectively. The DNA ladder used in the gel electrophoresis was tridye 2-log DNA ladder (New England Biolabs Inc. Ipswich, MA).*


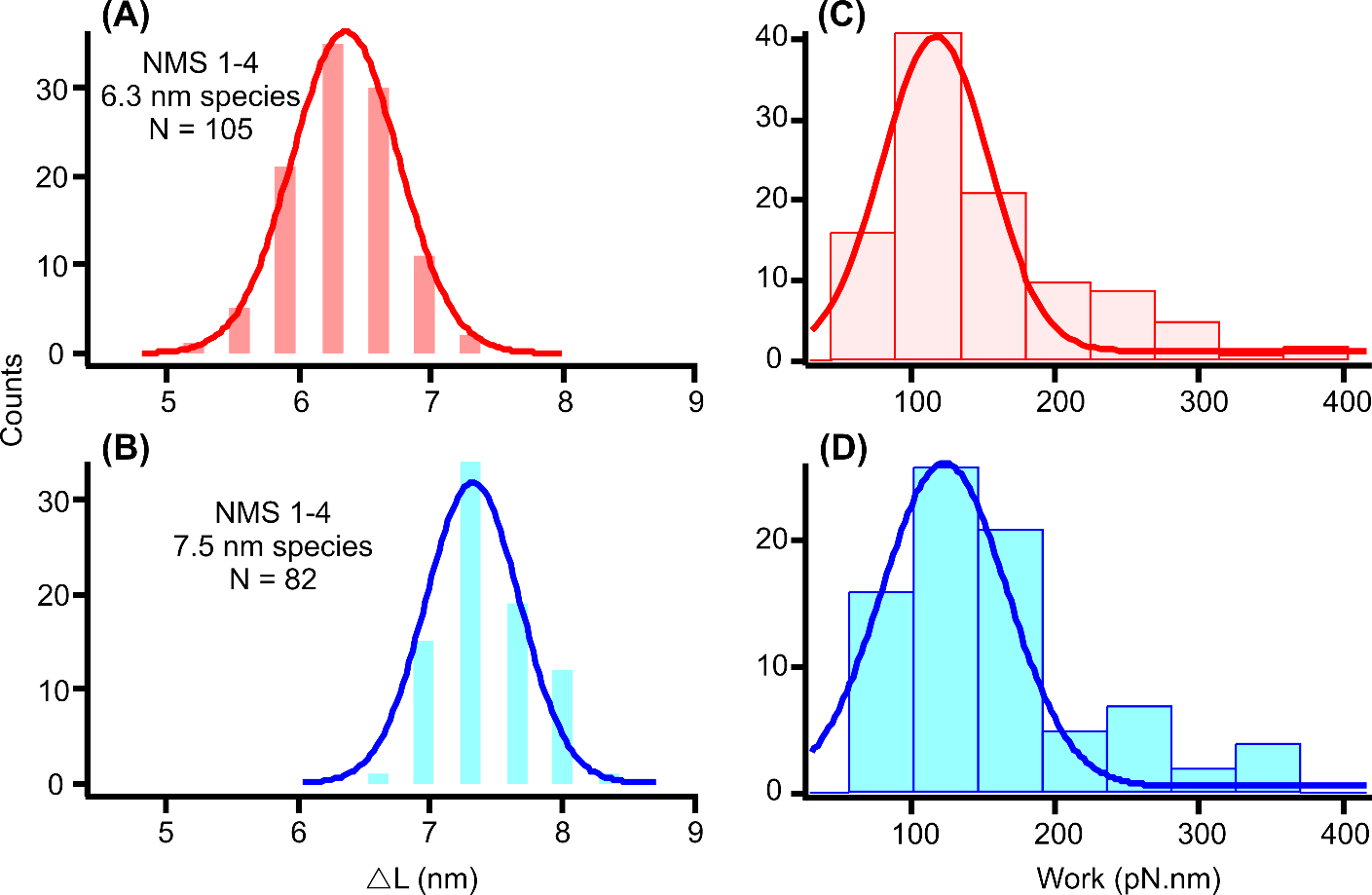


*Figure S5. Change-in-contour-length (ΔL, A and B) and work histograms (C and D) of the 6.3 and 7.5 nm populations observed during the unfolding of the structures formed in the construct NMS1-4 (see Figure 4A in the main text). The data corresponding to the two species were obtained through random deconvolution described in the Supporting Information.*


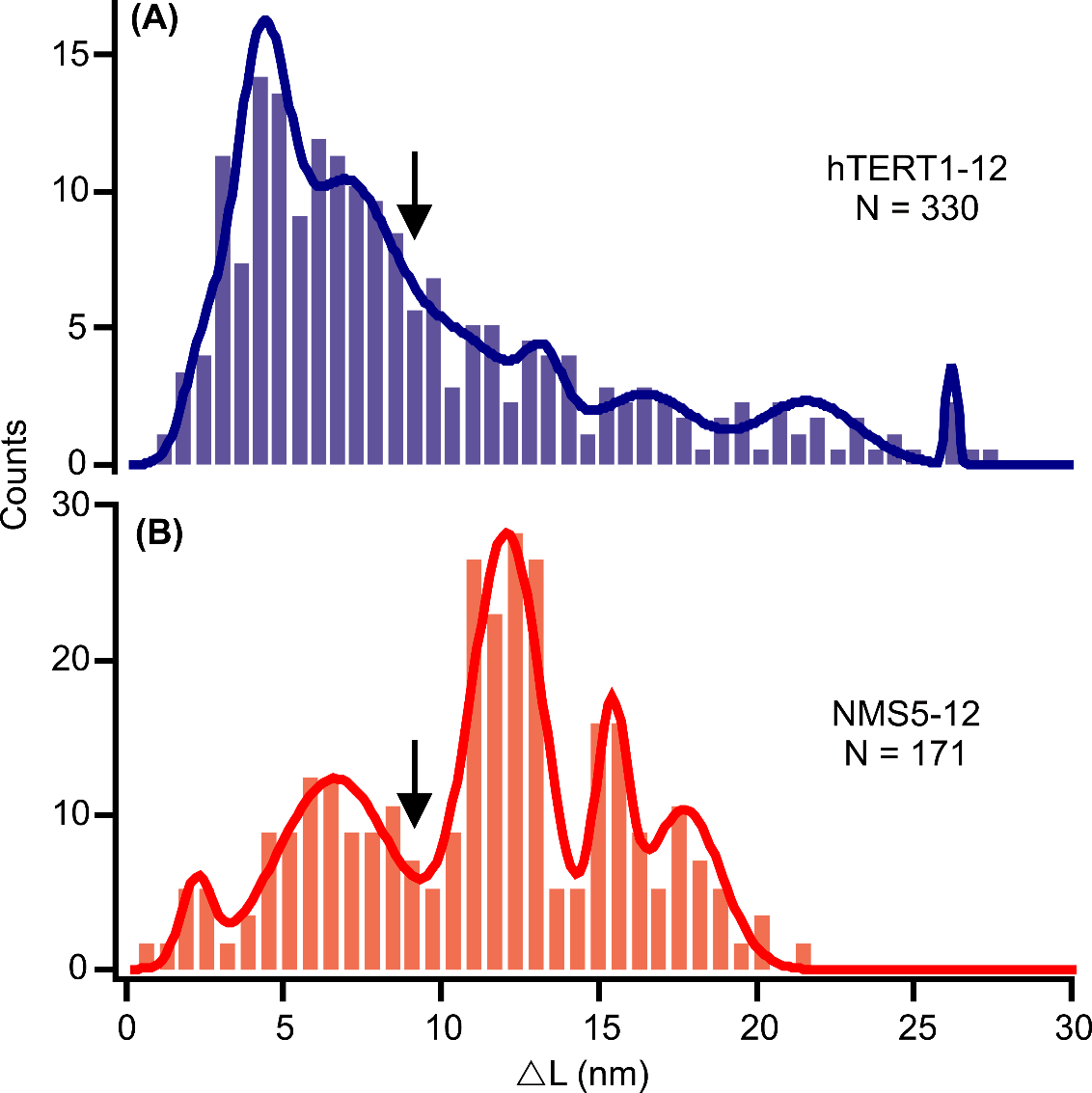


*Figure S6. Histograms of change-in-contour-length (ΔL) of the constructs (A) hTERT1-12 and (B) NMS5-12 (see Table S3 for a summary of ΔL measurement). The solid curves represent the multipeak Gaussian fitting using Igor Pro (WaveMetrics, Inc., OR). The arrows indicate the expected population (ΔL = 8.6 nm) of the middle G-quadruplex in a three G-quadruplex model proposed for the hTERT1-12 construct (5^th^ - 8^th^ G-tracts, see reference (*[*13*](#_ENREF_13)*)).*

*
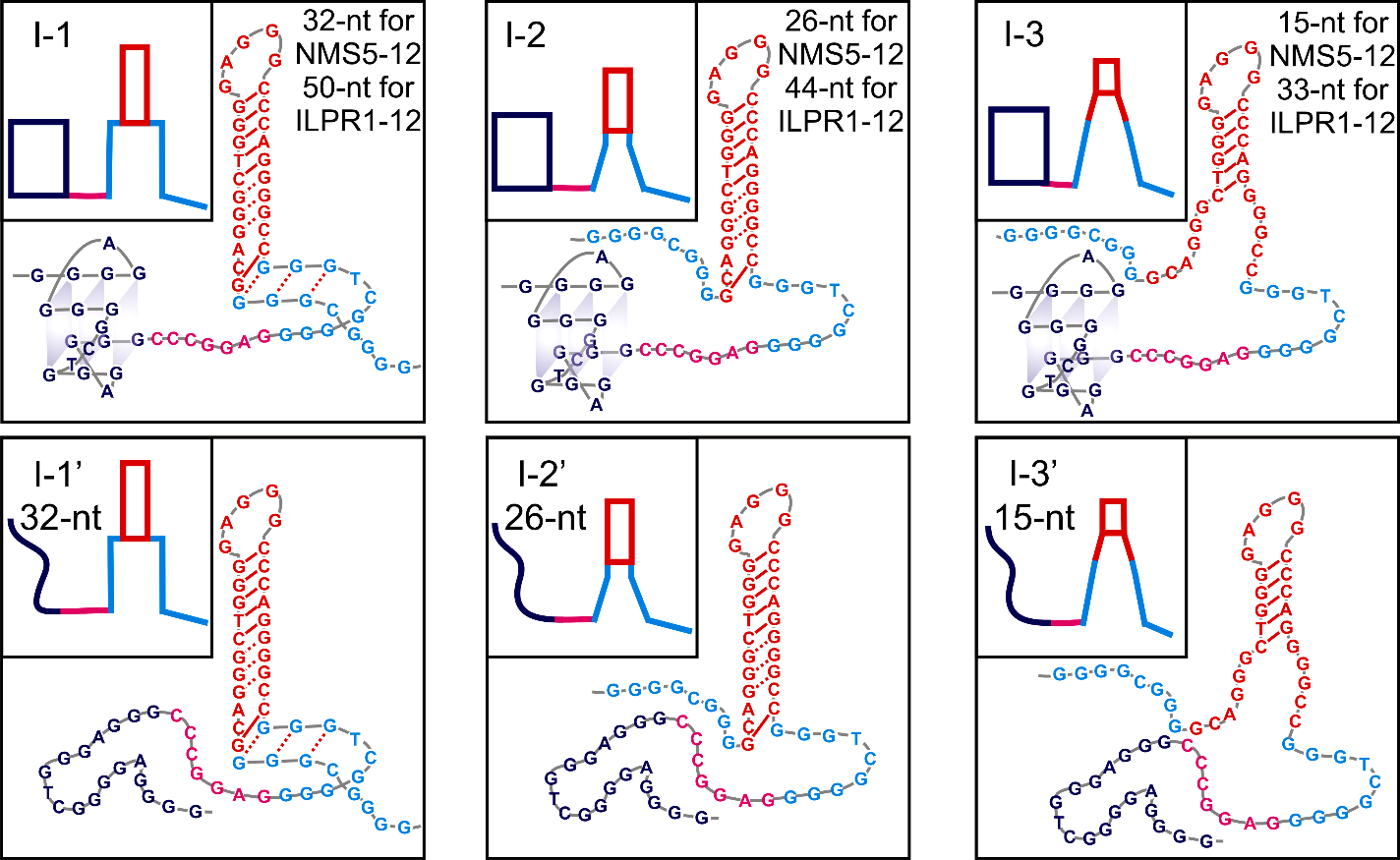
*

*Figure S7. Possible structures for intermediates during the unfolding of the NMS5-12 (I1, I2, and I3, see Figure 3) or the hTERT1-12 construct (I1, I2, I3, I1’, I2’, I3’ see Figure 6).*


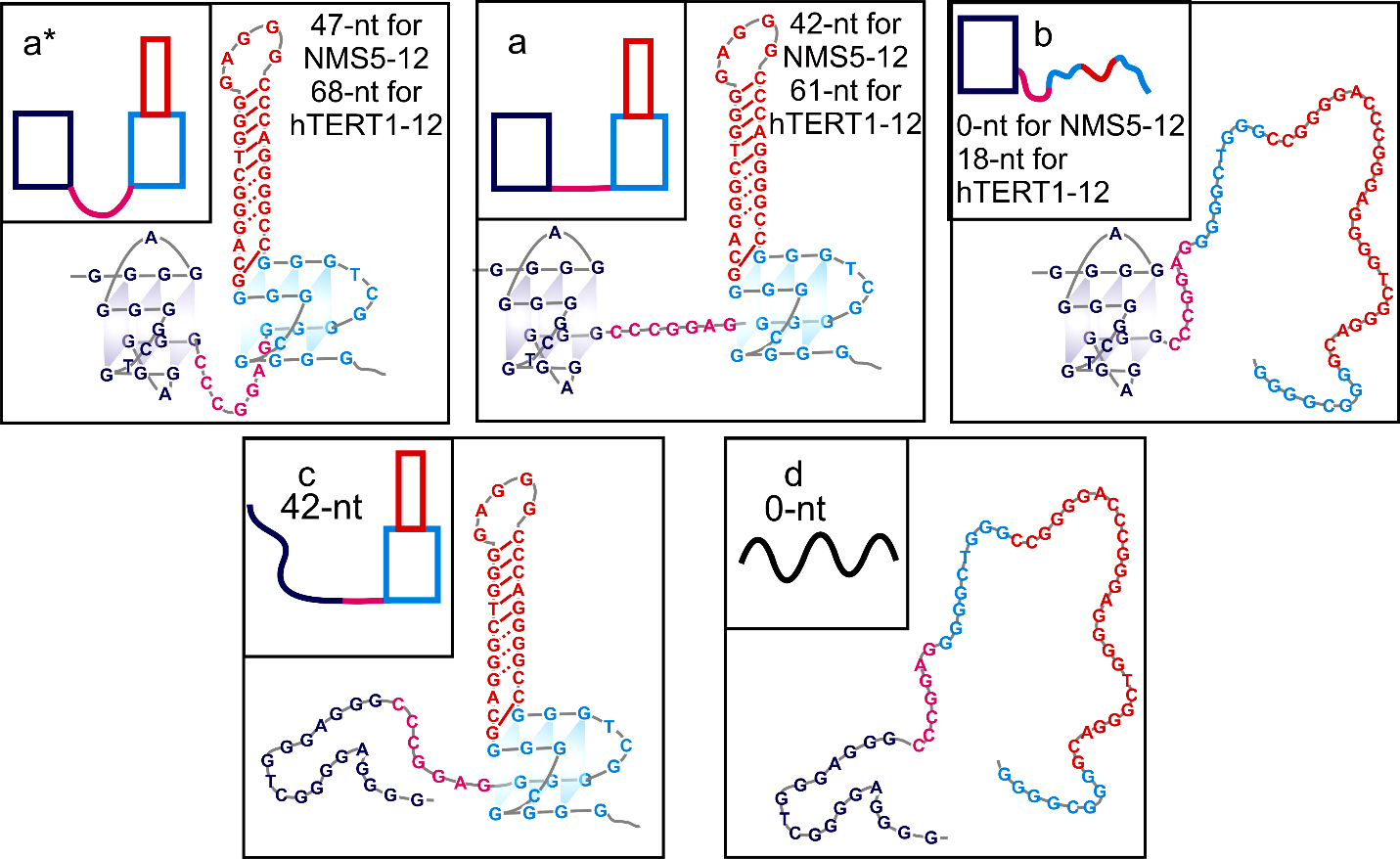


*Figure S8. Schematic drawing for possible structures in the main unfolding pathway of the NMS5-12 construct in Figure 3 (a*, a, and b) or the hTERT1-12 construct in Figure 6 (a*, a, b, c, and d).*

*
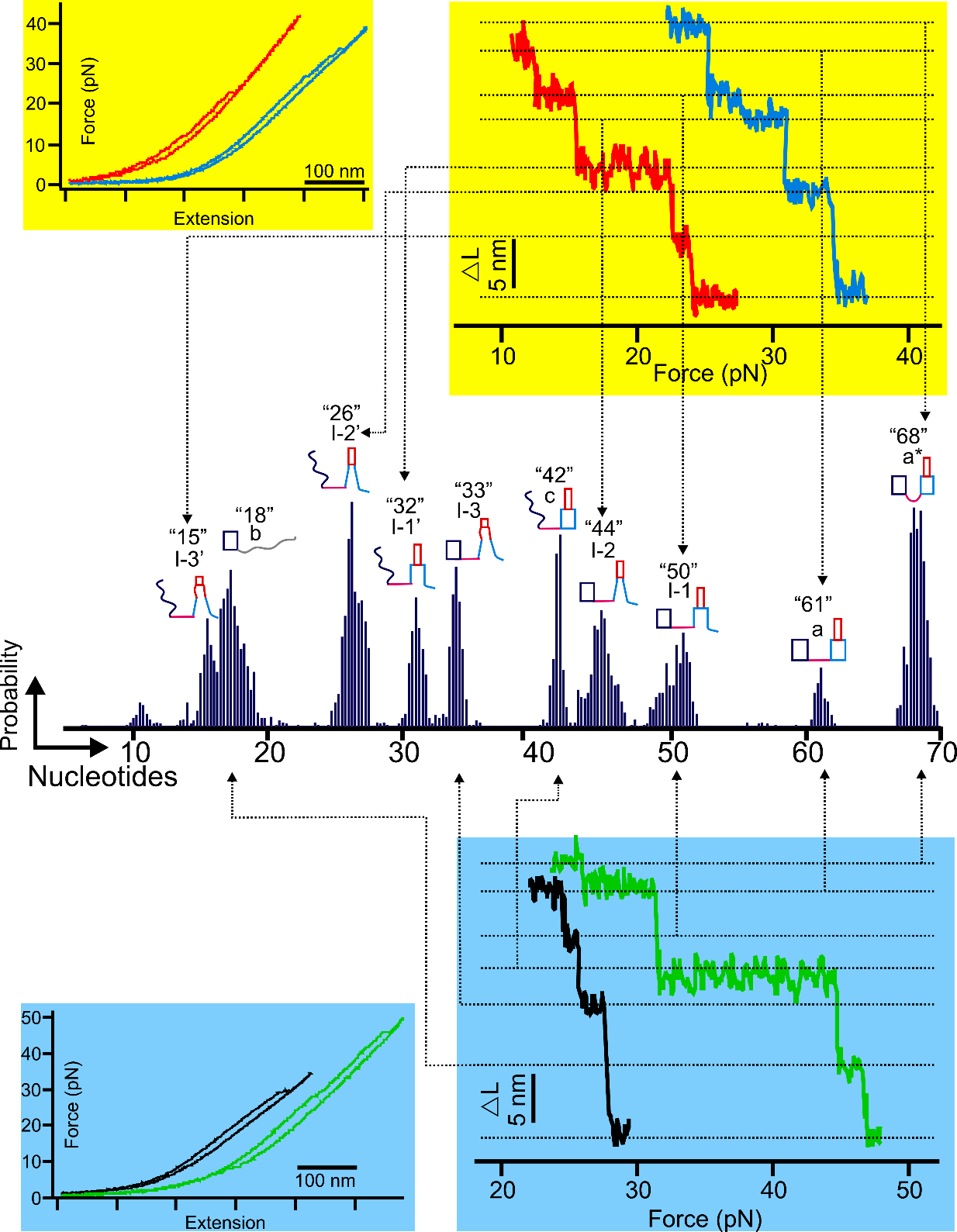
*

*Figure S9. Mechanical unfolding of the hTERT1-12 construct. (Top and Bottom) (left) Representative F-X traces observed during unfolding; (right) ΔL-F plots of the F-X curves. (Middle) Probability of observed populations (same as in Figure 5D). The numbers within quotations marks represent the number of nucleotides involved in a particular species. Schematic representation of the structure corresponding to each different population is given on top of each diagram (see detailed structures in Figures S6 and S7).*

**Supporting References**

1. Yu, Z., Schonhoft, J.D., Dhakal, S., Bajracharya, R., Hegde, R., Basu, S. and Mao, H. (2009) ILPR G-Quadruplexes Formed in Seconds Demonstrate High Mechanical Stabilities. *J. Am. Chem. Soc.*, **131**, 1876-1882.

2. Dietz, H. and Rief, M. (2004) Exploring the energy landscape of GFP by single-molecule mechanical experiments. *Proc. Nat. Acad. Sci. USA*, **101**, 16192-16197.

3. Yu, Z., Gaerig, V., Cui, Y., Kang, H., Gokhale, V., Zhao, Y., Hurley, L.H. and Mao, H. (2012) Tertiary DNA Structure in the Single-Stranded hTERT Promoter Fragment Unfolds and Refolds by Parallel Pathways via Cooperative or Sequential Events. *J. Am. Chem. Soc.*, **134**, 5157-5164.

4. Mills, J.B., Vacano, E. and Hagerman, P.J. (1999) Flexibility of single-stranded DNA: use of gapped duplex helices to determine the persistence lengths of poly(dT) and poly(dA). *J. Mol. Biol.*, **285**, 245-257.

5. Laurence, T.A., Kong, X., Jager, M. and Weiss, S. (2005) Probing structural heterogeneities and fluctuations of nucleic acids and denatured proteins. *Proc. Nat. Acad. Sci. USA*, **102**, 17348-17353.

6. Lim, K.W., Lacroix, L., Yue, D.J., Lim, J.K., Lim, J.M. and Phan, A.T. (2010) Coexistence of two distinct G-quadruplex conformations in the hTERT promoter. *J. Am. Chem. Soc.*, **132**, 12331-12342.

7. Shiflett, P.R., Taylor-McCabe, K.J., Michalczyk, R., Silks, L.A. and Gupta, G. (2003) Structural studies on the hairpins at the 3' untranslated region of an anthrax toxin gene. *Biochemistry*, **42**, 6078-6089.

8. Koirala, D., Mashimo, T., Sannohe, Y., Yu, Z., Mao, H. and Sugiyama, H. (2012) Intramolecular folding in three tandem guanine repeats of human telomeric DNA. *Chem. Commun.*, **48**, 2006-2008.

9. Dhakal, S., Schonhoft, J.D., Koirala, D., Yu, Z., Basu, S. and Mao, H. (2010) Coexistence of an ILPR i-Motif and a Partially Folded Structure with Comparable Mechanical Stability Revealed at the Single-Molecule Level. *J. Am. Chem. Soc.*, **132**, 8991–8997.

10. Jarzynski, C. (1997) Nonequilibrium Equality for Free Energy Differences. *Phys. Rev. Lett.*, **78**, 2690 - 2693.

11. Dhakal, S., Cui, Y., Koirala, D., Ghimire, C., Kushwaha, S., Yu, Z., Yangyuoru, P.M. and Mao, H. (2013) Structural and mechanical properties of individual human telomeric G-quadruplexes in molecularly crowded solutions. *Nucleic Acids Res.*, **41**, 3915-3923.

12. Cheng, W., Arunajadai, S.G., Moffitt, J.R., Tinoco, I., Jr. and Bustamante, C. (2011) Single-base pair unwinding and asynchronous RNA release by the hepatitis C virus NS3 helicase. *Science*, **333**, 1746-1749.

13. Chaires, J.B., Trent, J.O., Gray, R.D., Dean, W.L., Buscaglia, R., Thomas, S.D. and Miller, D.M. (2014) An Improved Model for the hTERT Promoter Quadruplex. *PLoS ONE*, **9**, e115580.

14. Palumbo, S.L., Ebbinghaus, S.W. and Hurley, L.H. (2009) Formation of a Unique End-to-End Stacked Pair of G-Quadruplexes in the hTERT Core Promoter with Implications for Inhibition of Telomerase by G-Quadruplex-Interactive Ligands. *J. Am. Chem. Soc.*, **131**, 10878-10891.
